# FKBP5 activates mitophagy by ablating PPAR-γ to shape a benign remyelination environment

**DOI:** 10.1038/s41419-023-06260-7

**Published:** 2023-11-11

**Authors:** Xingzong Sun, Menghan Qian, Hongliang Li, Lei Wang, Yunjie Zhao, Min Yin, Lili Dai, Hongkun Bao

**Affiliations:** 1 School of Medicine, Yunnan University, Kunming, 650091 China; 2 School of Agronomy and Life Sciences, Kunming University, Kunming, 650214 China

**Keywords:** Multiple sclerosis, Neuroscience

## Abstract

Multiple sclerosis (MS) is an autoimmune and neurodegenerative disease of the central nervous system (CNS) that is characterized by myelin damage, followed by axonal and ultimately neuronal loss, which has been found to be associated with mitophagy. The etiology and pathology of MS remain elusive. However, the role of FK506 binding protein 5 (FKBP5, also called FKBP51), a newly identified gene associated with MS, in the progression of the disease has not been well defined. Here, we observed that the progress of myelin loss and regeneration in Fkbp5^ko^ mice treated with demyelination for the same amount of time was significantly slower than that in wild-type mice, and that mitophagy plays an important regulatory role in this process. To investigate the mechanism, we discovered that the levels of FKBP5 protein were greatly enhanced in the CNS of cuprizone (CPZ) mice and the myelin-denuded environment stimulates significant activation of the PINK1/Parkin-mediated mitophagy, in which the important regulator, PPAR-γ, is critically regulated by FKBP5. This study reveals the role of FKBP5 in regulating a dynamic pathway of natural restorative regulation of mitophagy through PPAR-γ in pathological demyelinating settings, which may provide potential targets for the treatment of demyelinating diseases.

## Introduction

Multiple sclerosis (MS) is the most prevalent chronic inflammatory autoimmune disease of the central nervous system (CNS) and leads to demyelination, proliferation of astrocytes, microglial activation and axonal damage, resulting in movement, sensory, and cognitive loss [1]. MS affects more than 2.5 million people worldwide and generally affects young adults, with a prevalence of three female patients per male patient [2–4]. Although the precise etiology of MS remains unclear, it has been proposed to be caused by several factors, including a genetic predisposition to develop the disease and environmental agents that can stimulate the immune system and trigger an autoimmune reaction [5, 6].

The myelin sheath of the CNS is formed by oligodendrocytes surrounding the nerve axons, which can greatly accelerate the conduction of nerve impulses and nourish the nerve axons [7, 8]. Inflammation, toxicity, and other factors can damage oligodendrocytes and affect their normal function, leading to demyelination of the CNS and neurodegenerative diseases, such as MS [9, 10]. Characteristic pathological changes in MS include CNS nerve inflammation, demyelination, gliosis and neuronal loss [10]. In MS and its animal models, microglia are highly activated and participate in all phases of the disease [11]. Microglia are involved in both demyelination and remyelination phases of MS [12]. Microglia are highly activated in lesions of demyelination where they have been described to have both harmful roles, such as production of cytotoxic cytokines, contribution to oxidative damage, and promotion of pathogenic T cell responses, as well as beneficial roles, including promotion of oligodendrocyte differentiation and clearance of myelin and cellular debris [11, 13]. In MS patients with demyelinating diseases, oligodendrocyte precursor cells are activated, proliferate and migrate to the demyelinating lesion area, and differentiate into mature oligodendrocytes, eventually repairing the demyelinating lesions [14]. However, remyelination efficiency is substantially lower in patients with MS, and the survival and function of neurons are severely impaired in the absence of a myelin sheath, resulting in axonal degeneration and progressive deterioration of neural function [15]. The integrity of the myelin sheath after demyelinating injury and remyelination plays a key role in the recovery physiological functions of the CNS [4]. As the core of MS treatment, remyelination is a very promising research field in the treatment of MS; however, to achieve therapeutic remyelination, it is necessary to understand the relevant factors and the exact molecular mechanisms of remyelination [4, 16].

In cuprizone (CPZ)-induced acute demyelination models, not only myelin loss but also myelin regeneration and repair occur during this period [17]. CPZ binds to copper ions in the body, resulting in the loss of copper ions, which selectively act on the mitochondria of oligodendrocytes, leading to decreased expression of complex IV protein during mitochondrial respiration, resulting in mitochondrial dysfunction, and eventually oligodendrocyte apoptosis and myelin detachment [18]. Demyelination is accompanied by the expansion and transition of CNS-derived microglia and astrocytes to become more reactive. CPZ ingestion alters the mitochondrial function in the CNS of mice [19, 20]. In vitro, CPZ lowers the mitochondrial transmembrane potential of oligodendrocytes, but does not affect microglia, astrocytes, and neurons [21]. In vivo, three weeks of early CPZ administration results in morphological disturbances in oligodendrocyte mitochondria in the form of megamitochondria [20]. Enlarged megamitochondria are thought to reflect a compensatory response to elevated levels of ROS, based on evidence in vitro. After five weeks of the CPZ diet, mitochondria in the corpus callosum of treated mice swell and release mitochondrial cytochrome C and apoptosis-inducing factors, which contribute to impaired mitochondrial function [22]. Mitophagy, a highly conserved lysosome-dependent degradation pathway involved in the maintenance of cellular homeostasis, has been associated with MS [23]. Recent studies have shown that mitophagy, a process that selectively eliminates damaged mitochondria to regulate mitochondrial function and reduce oxidative stress, plays an important role in the pathogenesis of MS [24]. One of the features of MS is the presence of pathology-related oxidative stress and mitochondrial dysfunction. The PTEN-induced putative kinase 1 (PINK1) and Parkin signaling are key pathways in mitophagy control [25]. The PINK1-Parkin axis is involved in the regulation of the mitophagy process under MS-like conditions. Following mitophagy activation, Parkin is recruited from the cytosol into the outer mitochondrial membrane by PINK1.

FK506 binding protein 5 (also called FKBP51) is a 51-kDa FK506 binding protein that is a member of a family of immunophilins, FK506 binding proteins (FKBPs), and is involved in various biological processes, including immune regulation, protein folding, and transport [26]. As a chaperone protein, FKBP51 modulates the transcription factor nuclear factor kappa B (NF-κB), which alters the expression of proinflammatory cytokines [27]. In ionizing radiation-induced malignant melanoma, FKBP5 is associated with apoptosis resistance while inducing autophagy [28]. During antidepressant therapy, FKBP5 may be a potential target of some drugs related to autophagy [28]. Mitophagy is one of the molecular mechanism that has been linked to MS. FKBP5 levels in the cerebrospinal fluid and peripheral blood of patients with MS were significantly upregulated, suggesting that FKBP5 plays an important role [29, 30]. However, few studies have focused on mitophagy regulation of FKBP5 in demyelinating diseases. The current study has provided the first insight into the physiological function of FKBP5 in CPZ pathogenesis and describes the regulatory mechanism of FKBP5 in regulating a dynamic pathway of natural restorative regulation of mitophagy through PPAR-γ in demyelinating pathological settings. We discovered that FKBP5 is crucial for the progression of myelin loss and regeneration by mediating the activation of the PINK1/Parkin-mediated mitophagy pathway, in which the important regulator, PPAR-γ, is critically regulated by FKBP5, which facilitates the formation of a benign remyelination environment in the CNS.

## Results

### The degree of demyelination was significantly lower in FKBP5 deficiency mice than that of the wild type

**Fig. 1:**
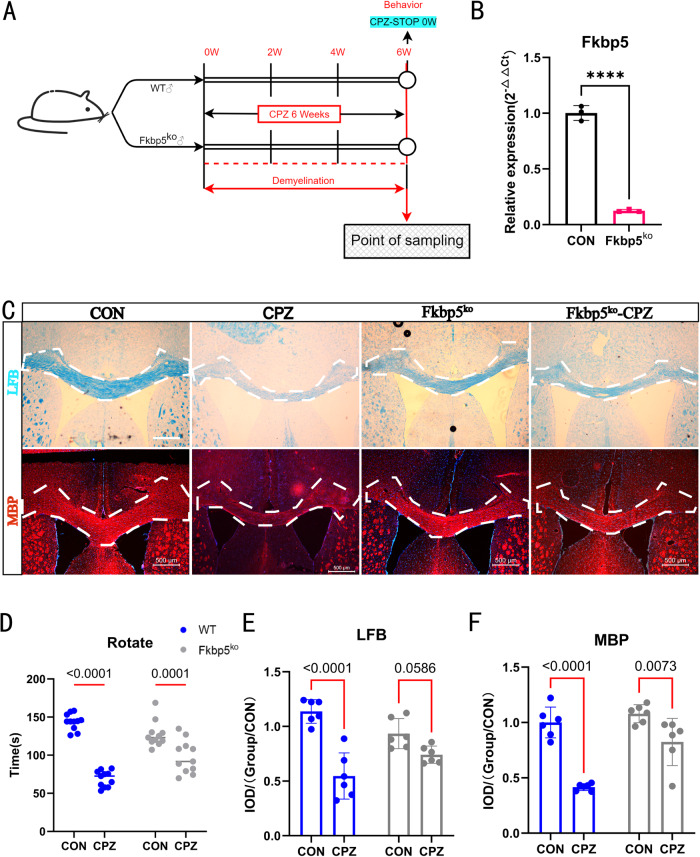
Fkbp5^ko^ mice had a weaker effect of simultaneous induction of demyelination compared to that in WT mice. **A** The degree of myelin loss in Fkbp5^ko^ mice were significantly lower than that in WT under the same conditions of CPZ induction model building flow chart. **B** RT-PCR examination of Fkbp5 expression levels in WT and Fkbp5^ko^ mice (*n* = 3, *t*-test). **C** Results of LFB staining and MBP fluorescence staining of brain tissue sections from blank-treated mice of WT or Fkbp5^ko^ and their corresponding CPZ model building mice. LFB staining and MBP fluorescence staining results analyzed by the Image J software for gray value of the white dotted box area. LFB staining and MBP fluorescence staining results for sections with a thickness of 20 μm, scale bars = 500 μm. **D** Behavioral statistics of rod turning experiments in mice with a fully automated rod turning machine speed of 30 rpm, each observation time of 3 min, and three observations per mouse for averaging (*n* = 11, two-way). **E**, **F** Separate staining for the two stains in **C**. IOD statistics were performed (*n* = 6, two-way).

To determine whether FKBP5 plays a role in the pathological environment under study, we designed a CPZ model using two mouse genotypes: wild-type (WT) and Fkbp5^ko^ (Fig. 1A). The efficiency of Fkbp5 knockout mice was demonstrated by RT-PCR (Fig. 1B). We verified the movement ability of the mice using rotarod test behavioral experiments (Fig. 1D), which showed a significant decrease in the movement ability of the WT-induced mice that also completed acclimatization training compared with the non-induced mice. Similarly, we also found that the CPZ group was less susceptible to staining when we stained the corpus callosum and periphery according to myelin morphological criteria. The results of Luxol Fast Blue (LFB) staining of the white matter (Fig. 1C) and fluorescent staining of myelin basic protein (MBP) (Fig. 1C) indicated a significant loss of myelin sheaths in CPZ-induced WT mice (Fig. 1E, F). However, the extent of myelin damage in Fkbp5^ko^ mice did not reach that in WT mice, suggesting that the deletion of FKBP5 may inhibit the loss of lesional myelin to some extent. These results showed that the morbidity rate of simultaneously induced Fkbp5^ko^ mice was lower than that of WT mice.

### FKBP5 plays a role in promoting demyelination and remyelination processes

**Fig. 2:**
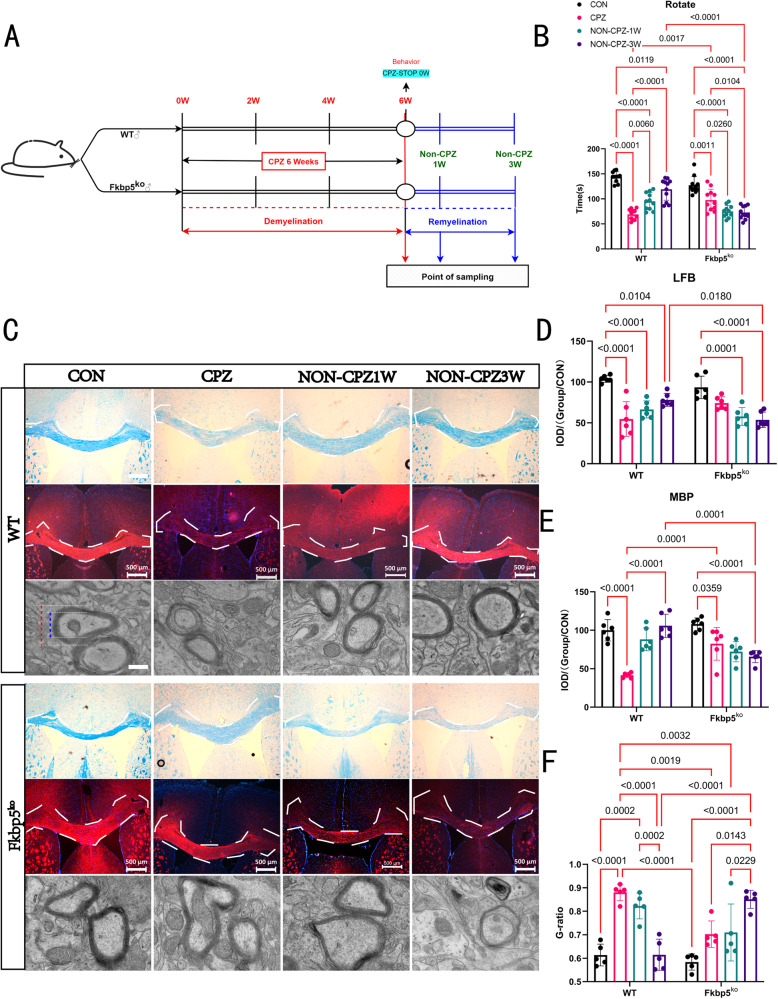
Fkbp5^ko^ mice do not recover well after the suspension of CPZ induction. **A** The remyelination process after myelin loss was simulated by combining the one- and three-week recovery groups to establish a flow chart of the remyelinated animal model. **B** Behavioral statistics of animal turning rods (*n* = 11) at the same instrumental parameters, 30 rpm, for 3 min. **C** LFB staining, MBP fluorescence staining, scale bars = 500 μm and transmission electron microscopy were examined for three-week health-recovery dynamics of myelin sheaths in the corpus callosum and surrounding tissue regions of mice of both genotypes, scale bars = 1 μm. **D**, **E** The statistics of each microscopic image shown in **C**, where **D**, **E** is the IOD statistic (*n* = 6, two-way). **F** The cell radius to myelin thickness ratio (G-ratio), as in the first electron microscope image in **C**, the blue/red line (*n* = 5, two-way).

To verify how FKBP5 affects the phenotype of mice during remyelination in conjunction with reversible myelin deficiency in CPZ model mice, we designed one- and three-week recovery groups based on a successfully established CPZ model mice (Fig. 2A) and compared the differences between WT and Fkbp5^ko^ mice during this dynamic change. Notably, based on the pre-turned rod behavioral experiment used to analyze changes in mice movement ability (Fig. 2B), consistent with the subsequent morphological analysis (Fig. 2C), we found that WT mice showed significantly stronger self-repairing effects, with progressively deeper white matter or MBP staining even during the three-week recovery period, which did not differ much from that of the control (CON) group. In contrast, Fkbp5^ko^ mice had a continued decline in their mobility after the loss of CPZ induction (Fig. 2B). The color of the target area gradually became lighter, indicating a continuous decrease in the coverage of myelin and MBP (Fig. 2D, E), and finally exhibited the same degree of myelin loss during the three-week recovery period as that in WT mice at the beginning of the modeling process, a phenomenon that was simultaneously observed in the corpus callosum of the various groups of mice by transmission electron microscopy (TEM) (Fig. 2C). The calculated G-ratio (Fig. 2F), indicated that the thickness of myelin sheaths was thinning with the extension of the induction time in Fkbp5^ko^ mice, which suggests that the deletion of FKBP5 slowed down the overall development of the pathologic milieu. Collectively, these data suggest that *FKBP5* deletion inhibits the efficiency of myelin loss and regeneration; that is, the development of the pathological environment is delayed.

### FKBP5 deficiency causes abnormal responses of microglia and astrocytes in dynamic repair

**Fig. 3:**
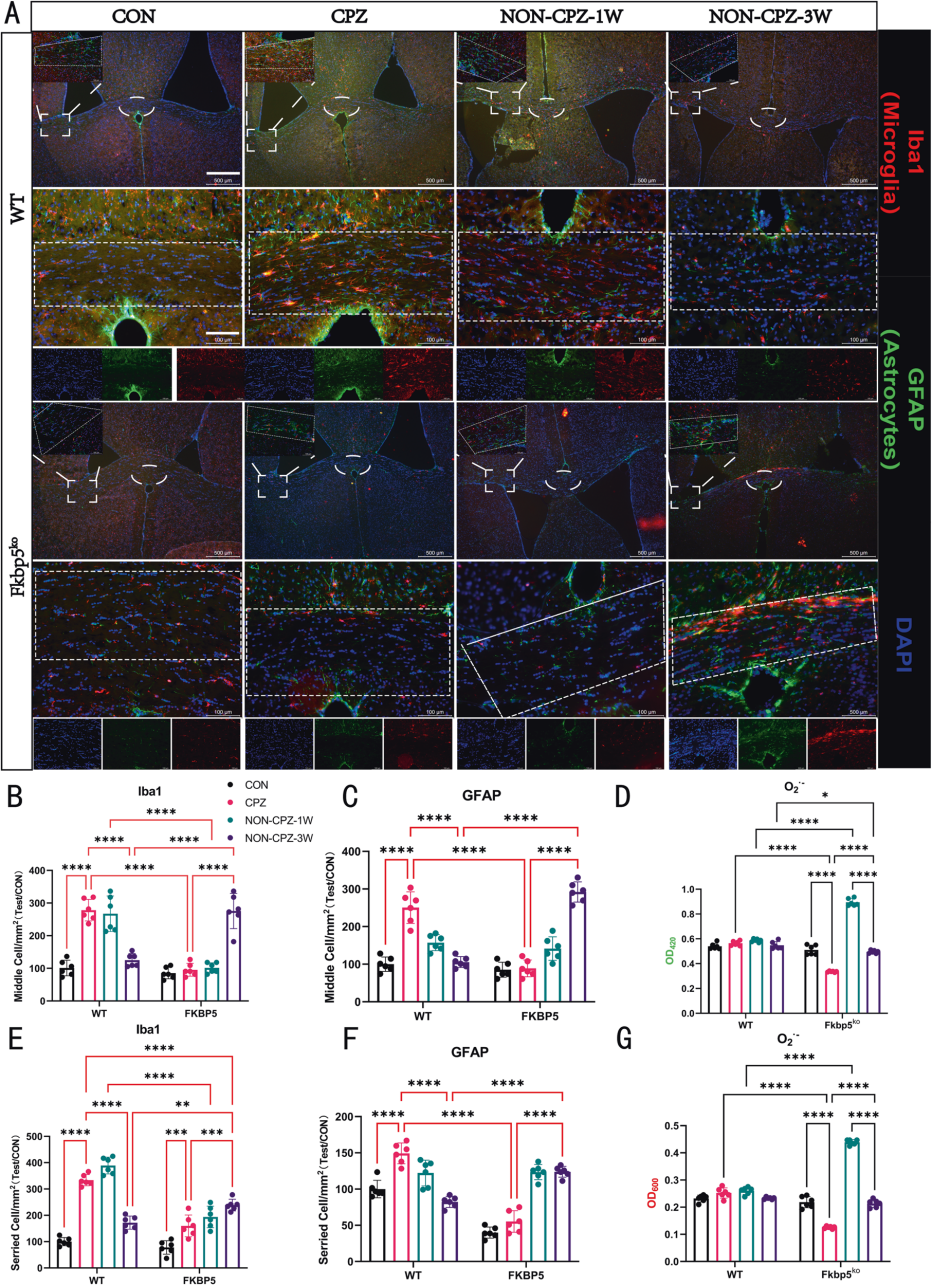
FKBP5 deletion resulted in delayed microglia and astrocyte enrichment processes. **A** Fluorescence staining maps showing changes in microglia (red) and astrocytes (green) in the corpus callosum and surrounding tissue regions of brain tissue in WT and Fkbp5^ko^ mice at 0, 1, and 3 weeks after induction of demyelination. Two regions were identified as glial cell enrichment areas by low magnification observations. The lateral horn of the ventricle is located in the region of the corpus callosum (shown by the dashed box) and the central region of the coronal section of the corpus callosum (dashed oval box), and both are framed in the region of the corpus callosum at high magnification. **B**, **C**, **E**, **F** Histograms of the number of positive microglia and astrocytes at the two locations, with the two arms in the downward column and the central location in the upward column. **D**, **G** Equal masses of fresh tissues from each group were cut up, treated with trypsin, and then detected at two wavelengths of 420 and 600 nm for superoxide anion (O_2_^▪-^) content and counted, respectively.

To further demonstrate the changes in the demyelinating microenvironment, we investigated the dynamics of microglia and astrocytes (Fig. 3A). We counted the number of positive cells for the microglia marker protein, Iba1 (Fig. 3B, E), and the astrocyte marker protein, GFAP, in two different regions (Fig. 3C, F). The first one was located in the center of the corpus callosum, and the second in the two arms of the corpus callosum and ventricle junction. The above two regions were observed as glial cell-enriched zones under low magnification. Morphological studies in Fig. 3A showed that WT mice were significantly enriched in microglia and astrocytes in the lesion area after completion of CPZ modeling and gradually returned to the resting state over time (Fig. 3A). Fkbp5^ko^ mice, which had also completed CPZ modeling, did not show any enrichment of microglia or astrocytes in the observed area. This phenomenon persisted until the third week after the abolition of CPZ treatment (Fig. 3A), when we found that microglia and astrocytes normalized in WT mice, but showed an abnormal increase in Fkbp5^ko^ mice, a trend that was consistent with all stages of myelin loss.

High concentrations free radicals have been found in inflammatory MS lesions [31]. A study showed that increased generation of superoxide anion (O_2_^▪-^) in the blood stimulated with phorbol myristate acetate (PMA) has been found in MS patients [32]. Based on preliminary experiments, we focused on the shaping of myelin damage and the repair environment, and investigated fresh tissue O_2_^▪-^, which is a strong oxidizing anion that is produced under stressful conditions in tissue cells and causes a high degree of damage to the organism. After detecting the degrees of response at 420 and 600 nm and conducting a statistical analysis (Fig. 3D, G), we were surprised to find that both mice genotypes showed a peak in superoxide anion after one week of recovery. Unlike WT mice that declined after slow growth, Fkbp5^ko^ mice produced a significant decrease in O_2_^▪-^ under the CPZ modeling and a significant increase after one week, suggesting that FKBP5 may influence the natural onset of a range of oxidative processes occurring within the cell.

### Transcriptome analysis suggests the association of FKBP5 with PPAR-γ and its regulatory role in mitochondria

**Fig. 4:**
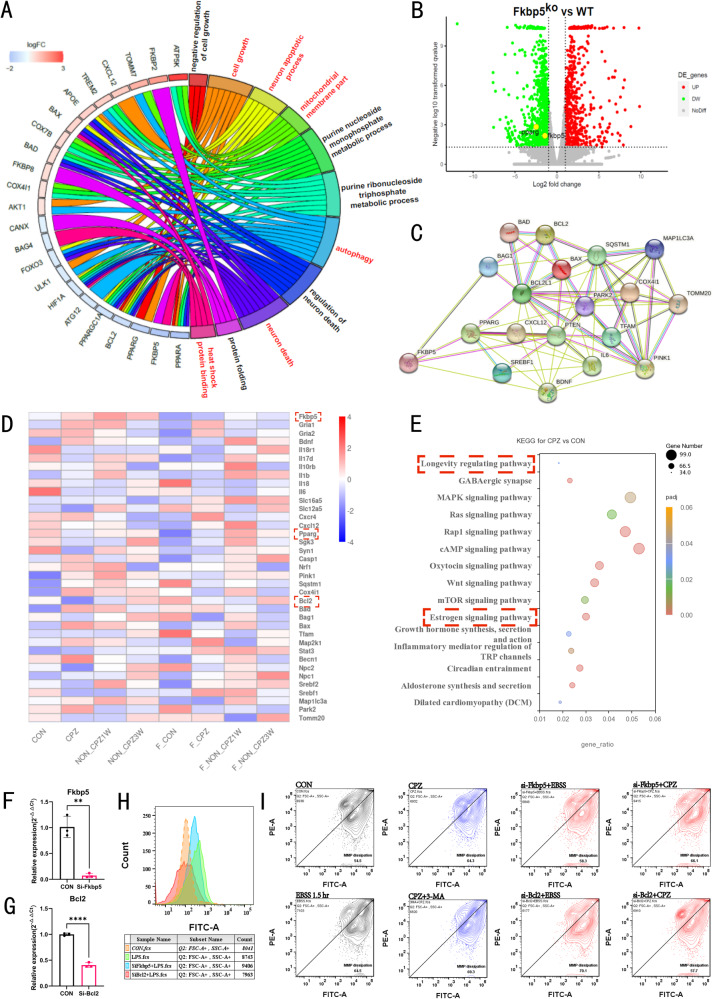
Enrichment analysis of genes related to the regulation of Fkbp5 and mitochondrial activity and construction of a protein interaction network to determine the feasibility of Fkbp5 regulating mitochondria through Pparg. **A** GO pathway enrichment analysis, including multiple pathways of cellular autophagy, mitophagy and cellular metabolism. **B** Volcano plot showing the changes in gene expression in the WT mice vs. Fkbp5^ko^ mice. Green represents downregulation, and red represents upregulation. **C** PPI analysis of the FKBP51 interactions with mitophagy-associated proteins. **D** Heatmap analysis of differentially expressed genes associated with mitophagy between groups, with stronger color biases toward blue indicating lower expressions and stronger biases toward red indicating higher expressions. Membrane-making had the opposite trend of Fkbp5 expressions to that of Pparg and Bcl2, and the same as Pink1. **E** Bubble plots of the KEGG enrichment analysis in the WT mice blank vs. model group. Bubble sizes represent the numbers of enriched genes, where Fkbp5 was enriched in the estrogen signaling pathway and Pparg was enriched in the longevity regulating pathway (marked by red box), *p* < 0.05. **F**, **G** Showing RT-PCR results of Fkbp5 and Bcl2 interference by siRNA in primary brain tissue cultures, respectively (*n* = 3, one-way). **H** Flow cytometry in the FITC green fluorescence band validated the results of ROS production in different subgroups, showing a relative role for Fkbp5 versus Bcl2. **I** MMP under starvation (EBSS treatment for 1.5 h) conditions by JC-1, showing a significant decrease in MMP in the Bcl2-interfered group, while the Fkbp5-interfered group improved relative to the CON+EBSS group.

To determine through which possible pathways FKBP5 affects the physiological process of remyelination in organisms, we sequenced the transcriptome of the available tissue samples, and a gene ontology (GO) analysis showed (Fig. 4A) that FKBP5 is strongly associated with autophagy-related genes and mitochondria-mediated energy metabolism. Based on a CPZ modeling Kyoto Encyclopedia of Genes and Genomes (KEGG) analysis (Fig. 4E), FKBP5 was enriched in the estrogen signaling pathway, which would has a significant regulatory effect on Bcl2 (Fig. 4D). The current mainstream view is that Bcl2, as a classical cytoprotective factor, inhibits autophagy [33], whereas Fkbp5 shows the opposite trend to Bcl2 in the development of the pathological environment, suggesting that it may play a role in promoting autophagy. To verify this hypothesis, we used primary cultures of rat brain tissue to simulate the in vivo environment and manipulate Fkbp5 and Bcl2 using siRNA (interference efficiency validation is shown in Fig. 4F, G, and Supplementary Fig. S1B, C). Lipopolysaccharide (LPS) induction was applied for 24 h, and reactive oxygen species (ROS) signals were detected using flow cytometry (Fig. 4H). The two types of genes also showed opposite effects. Subsequently, we wished to verify the ability of Fkbp5 and Bcl2 to modulate mitochondrial membrane potential (MMP) under starvation (EBSS treatment for 1.5 h) conditions by JC-1 to further clarify the role of genes in mitochondrial function. The results showed a significant decrease in MMP in the Bcl2-interfered group, while the Fkbp5-interfered group improved relative to the CON group (Fig. 4I). Importantly, we added 3 mM CPZ for 24 h, with the aim of verifying whether CPZ could play a role in promoting mitochondrial damage in in vitro experiments, and found its value in inducing MMP reduction and its pathogenesis was more strongly dependent on Fkbp5 than on Bcl2. However, the addition of the autophagy inhibitor, 3-MA, further exacerbated the process (Fig. 4I). Therefore, Fkbp5 may play an important role in inducing myelin desquamation via CPZ in vivo. We analyzed the genes detected in the Fkbp5 gene-deficient group versus the WT group (Fig. 4B); the volcano plot showed that knock out of FKBP5 was accompanied by a decrease in PPAR-γ expression. Notably, heatmap analysis of the whole group showed that the upregulation of FKBP5 due to CPZ modeling (Fig. 4D) resulted in a concomitant decrease in PPAR-γ expression. Therefore, we used FKBP5-PPAR-γ-Bcl2 as the core and spread outward to search for possible key nodes between CNS demyelinating diseases and mitophagy, and tested the expected accuracy by subsequent experiments. Based on the screening results, in the demyelinating environment, the increase of FKBP5 caused by pathological environmental changes rather than the deletion of the *FKBP5* gene in the natural state was associated with the decrease of PPAR-γ. Additionally, the change of FKBP5 was inversely related to the change of Bcl2. We plotted a protein-protein interaction (PPI) network analysis (Fig. 4C) that found that FKBP5-PPAR-γ could crosstalk well with the mitophagy pathway. To verify the association between FKBP5 and PPAR-γ and how it regulates the autophagy-mitophagy process and affects mitochondrial function and dynamics, we performed the following experiments.

### In vitro experiments reveal that Fkbp5 maintains mitochondrial activity in pathological states by inhibiting Pparg

**Fig. 5:**
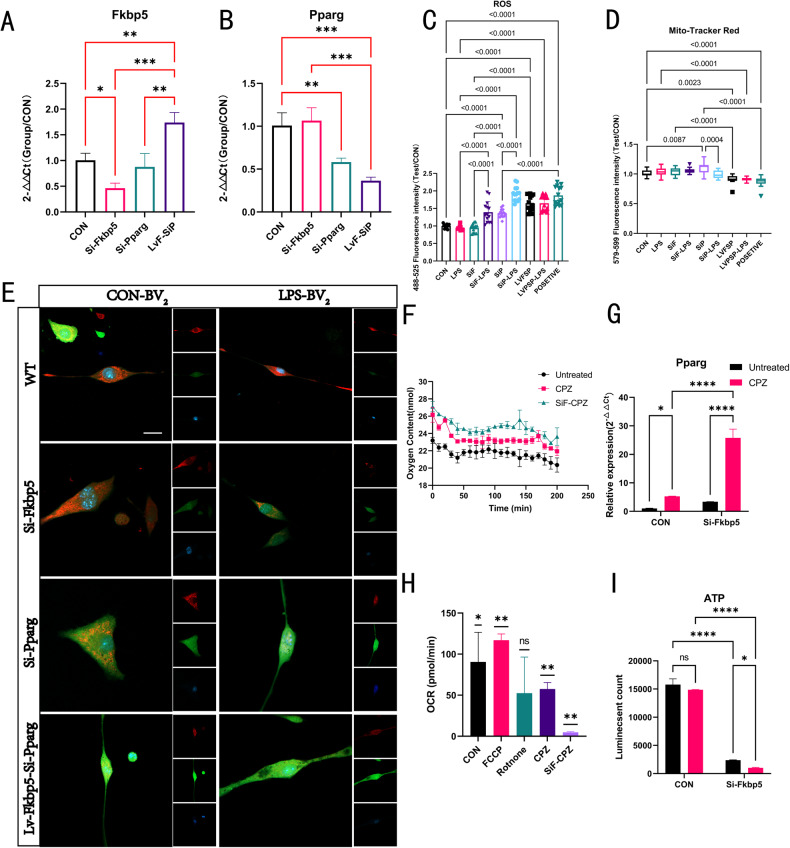
In vitro experiments were performed to validate the biological activity of Fkbp5 in inhibiting Pparg in an inflammatory environment and CPZ induction. **A**, **B** The results of RT-PCR tests on RNA extracted after incubation for more over 48 h in four groups corresponding to the subsequent experimental base groupings of blank treatment, interference with Fkbp5, interference with Pparg, and simultaneous LV infection overexpressing Fkbp5 and interference with statistical plots (*n* = 3, one-way). **C**–**E** Staining results of active mitochondria and ROS, with ROS fluorescently labeled in green and active mitochondria fluorescently labeled in red, in nine groups in a mouse-derived microglial cell line (BV2) based on interference with FKBP5 and PPAR-γ; overexpression of FKBP5 and interference with PPAR-γ were observed on the basis of these two genes on cells in LPS after 6 h of induction, and Rosup (a class of drugs that stimulate the production of ROS) was designed to treat the stimulated cells as a positive control, which is located in the upper left corner of the CON group. Fluorescence readings of ROS and active mitochondria were collected and statistically analyzed between groups using a fluorescent zymograph in the 488–525 nm and 579–599 nm bands, respectively (*n* = 14, one way). **F** The oxygen content of each subgroup of primary brain tissue cultures in the last 200 min was recorded after 24 h of treatment with 3 mM CPZ as verified by the Oxygen Probe fluorescence reaction, with a test interval of 10 min and a detection band at 500–650 nm (*n* = 3). **G** Verification of Pparg expression levels under CPZ induction by RT-PCR in primary brain tissue cultures (*n* = 3, two-way). **H** Calculation of OCR per unit time for each group based on Figure F data (*n* = 3, *t*-test). **I** Luminescent ATP kit to detect ATP production after 4 h of CPZ treatment in each primary brain tissue culture group (*n* = 3, two-way).

To verify the interaction between FKBP5 and PPAR-γ on mitophagy in the context of pathological stimuli, we designed the following experiments using a mouse microglia cell line (BV2) as a template for Fkbp5-siRNA-interfering (SiF), Pparg-siRNA-interfering (SiP), and Fkbp5 overexpression and Pparg interference (LVFSP) subgroups. LPS-induced short-term inflammatory subgroups (-LPS) were added for 6 h during each stable transfection or infection time period. ROS production and mitochondrial activity were quantified for the above subgroups (Fig. 5C, D), and results of gene manipulation verified using RT-PCR (Fig. 5A, B). After induction of the LPS neuroinflammatory response in each group, the fluorescence intensity of ROS and active mitochondria after 6 h of induction were detected. We found that interference with Fkbp5 did not affect ROS production in cells under non-LPS induction, but LPS induction resulted in large amounts of ROS production without affecting mitochondrial activity (Fig. 5C–E). This suggests a facilitative effect of FKBP5 on mitophagy, while the SiP group generated large amounts of ROS by LPS induction. This phenomenon remained evident even in the non-induced group (Fig. 5E), whereas a significant reduction in the number of active mitochondria was observed in this group under inflammatory induction (Fig. 5E), implying a protective effect of PPAR-γ on mitochondrial survival. Based on the above conjecture and the fact that lentiviral vectors (LV) infection causes FKBP5 overexpression and interferes with the PPAR-γ subgroup, we found a natural trend of reduced active mitochondria in this group (Fig. 5E). When FKBP5 was upregulated, PPAR-γ downregulation increased in natural state ROS. However, the LPS-induced state did not continue to increase ROS production, and the active mitochondria were slightly lower than those the other subgroup of cells.

In the CNS, oligodendrocyte metabolism and axonal myelination depend on their relationships with other nerve cells. Hence, we tested the effect of CPZ on autophagic recruitment in a mixed glial cell (MG) culture in which oligodendrocytes were grown on a layer of astrocytes and neurons. MS in the active phase inhibits the mitochondrial activity, thereby triggering mitophagy. Therefore, we interfered with Fkbp5 expression using siRNA in primary brain tissue cultures for 48 h, followed by CPZ treatment for 24 h. We recorded the oxygen content of the culture medium 200 min before the end of the incubation (Fig. 5F) and calculated the oxygen consumption rate (OCR) per unit time. We found that CPZ induced a decrease in OCR, whereas deletion of Fkbp5 significantly inhibited OCR (Fig. 5H). To further test the effect of Fkbp5 on mitochondrial dynamics, we examined ATP production 4 h after CPZ application. We tested ATP production after the application of CPZ for 4 h, and these data corroborated the OCR results (Fig. 5I). RT-PCR analysis of Pparg in samples 24 h after CPZ induction revealed that Pparg was abundantly expressed in the CPZ-treated group in the absence of Fkbp5 (Fig. 5G), which further clarified the inhibitory effect of Fkbp5 on Pparg.

### FKBP5 directly acts and inhibits PPAR-γ in vivo

**Fig. 6:**
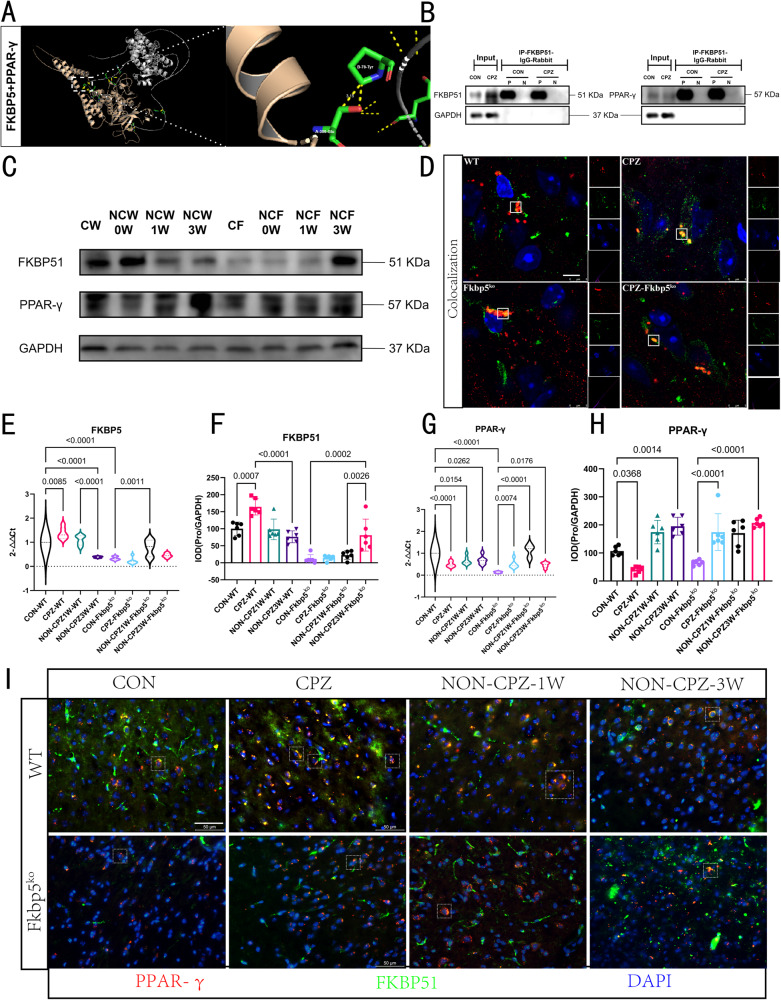
FKBP51 directly interacts with PPAR-γ and inhibits the latter from acting. **A** Visualization of the rigid molecular simulations of docking between FKBP51 and PPAR-γ. free energy is below zero, so spontaneous docking is considered. **B** CoIP results pulled with mouse-derived FKBP51 antibody indicate an interaction between the two proteins. “input” results indicate non-IP WB bands, both proteins are detectable, and negative control results “N” signal. **C** Protein blots showing FKBP51 and PPAR-γ results, with GAPDH selected as the internal reference protein. **D** Microscopic cell maps taken using confocal for each genotype and its model group, box marks indicate overlapping regions of proteins and co-localization results (WT, CPZ, Fkbp5^ko^, Pearson’ coefficient R values and Manders’ coefficients for CPZ-Fkbp5^ko^ were 0.49 and 0.878; 0.85 and 0.978; 0.58 and 0.929; 0.86 and 0.960, respectively). **E**–**H** shows WB and RT-PCR statistics for the two classes of proteins, respectively, with no differences between labeled groups indicating no statistical differences (*n* = 6, one-way). **I** Results of immunofluorescence of two types of proteins, green fluorescence points to FKBP51 and red to PPAR-γ, showing the results of co-staining immunofluorescence of two types of proteins.

To further demonstrate whether there is a direct interaction between FKBP5 and PPAR-γ, we did molecular simulation docking visualization analysis with the two types of proteins of human origin (Fig. 6A). The calculated free energy of rigid docking between the two proteins was −7.5 kcal/mol, suggesting that there is a structural basis for direct interaction between the predicted models of the two types of proteins. However, the subcellular localization analysis of the two types of proteins revealed the possibility of intersection during the total process of cell development, suggesting that there is a spatial basis for the mutual regulation between them (Supplementary Table S1). Then, we verified the biological basis of the association of the two types of proteins at the molecular level using co-immunoprecipitation (CoIP) (Fig. 6B), and found that PPAR-γ and FKBP5 could bind to each other in the total proteins. The co-localization coefficients of FKBP5 and PPAR by immunofluorescence-γ co-localization coefficients were in the confidence intervals, which fully demonstrated that the binding of the two types of proteins was stable and spontaneous. The fluorescence signals of these two proteins showed a high overlapping trend with the CPZ modeling (Fig. 6D, I). These findings suggest that FKBP5 can have a regulatory effect on PPAR-γ and this regulatory process is in the CPZ-induced demyelination model. Immediately following our molecular biology experiments on tissue samples (Fig. 6C), we found that FKBP5 upregulation in WT mice were accompanied by a decrease in PPAR-γ expression upon demyelination, whether validated at the RNA level (Fig. 6E, G) or the protein level (Fig. 6F, H). Notably, we searched the transcriptome sequencing results for members coregulated with the PPAR family and found that the trend of PPAR-γ activators, including PGC1-α, did not change significantly with Fkbp5 knockdown (Supplementary Fig. S2). Here we speculated that since FKBP5 expression never reached the level of WT mice, it is difficult to inhibit the activity of PPAR-γ, which is also stimulated by the demyelinating pathological environment.

### FKBP5 accelerates autophagy in tissue cells in a demyelinating environment in vivo

**Fig. 7:**
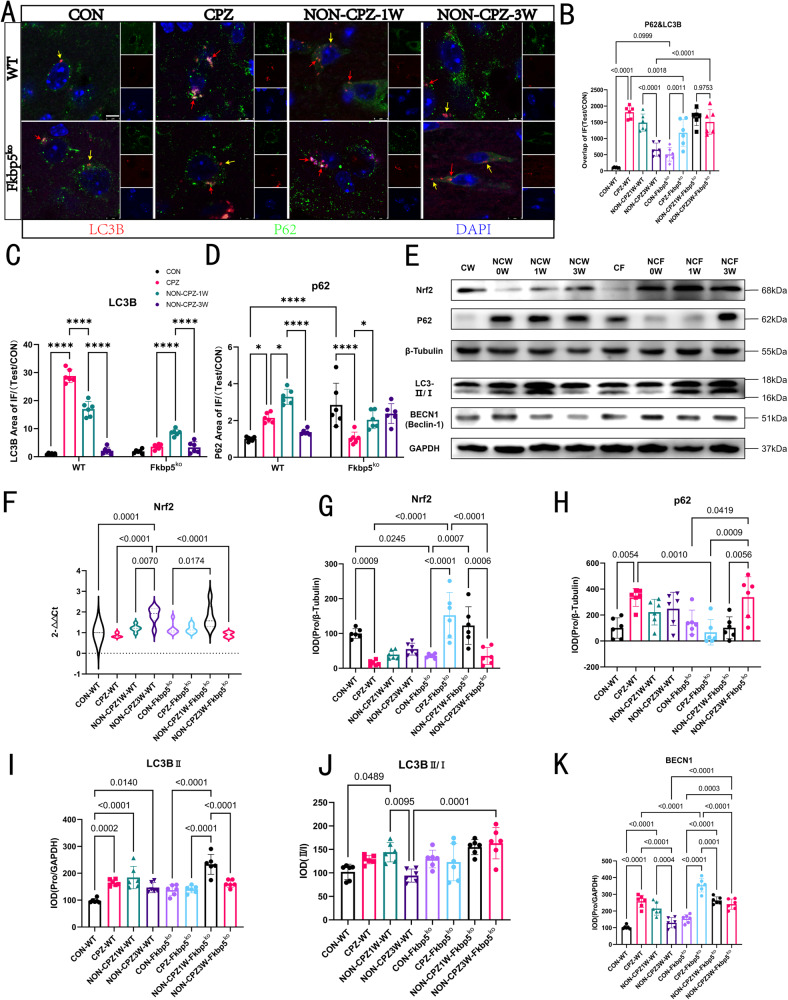
FKBP5 deficiency constrains autophagy. **A** P62 and LC3B in mice coronal section callus and surrounding tissues. the images show the results of laser confocal single cell images, scale bars = 5 μm, red arrows mark the nonoverlapping areas and yellow arrows mark the overlapping areas indicating the part of autophagy occurrence. **B** Demonstration of overlapping areas (*n* = 6, one-way). **C**, **D** Shows the fluorescence intensity statistics of two proteins, LC3B in the downward column of the bar graph direction and P62 in the upward column (*n* = 6, two-way). **E** Autophagy regulation of the upstream nuclear protein Nrf2, autophagy substrate protein, P62, and autophagy effector proteins, LC3B and Becn1. Protein blotting results with β-tubulin as the internal reference. **F** RT-RCR results for Nrf2 (*n* = 3, one-way). **G**–**K** Statistical results of the IOD values for the above four proteins (*n* = 6, one-way).

Before performing the in vivo autophagy-related studies, we performed in vitro experiments targeting CPZ induction treatment to primary brain tissue cultures (Supplementary Fig. S3A), indicating oligodendrocytes were more significantly affected than astrocytes and microglia. The transcriptomic-based hints that we successively analyzed the effects on autophagy and mitophagy in animal tissues based on FKBP5 knockout, which necessarily occur during the development of a CPZ-induced pathological environment. By analyzing SQSTM1/p62 with LC3B (Fig. 7A), a graphical representation of the results used for statistical purposes is shown in Supplementary Fig. S4A. We found that the WT mice showed an overall deepening to gradual recovery of autophagy levels, whereas FKBP5^ko^ mice did not return to normal levels during the same three-week recovery period (Fig. 7C, D). Our separate detection of the fluorescence intensities of the two types of proteins (Fig. 7B) also showed that activation of the autophagic process in WT mice was significantly more intense. We then observed the expression levels of Nrf2, SQSTM1/p62, LC3B, and BECN1 at the molecular level (Fig. 7E) and found that in the Nrf2 statistical data, FKBP5 mice appeared to be moving toward autophagy inhibition compared with the WT group, where autophagy levels first increased and then decreased over time (Fig. 7F, G); although the degree of this inhibition was always at modest levels. However, the two were not found to be synergistic in the p62 statistical data (Fig. 7H), which is a phenomenon found in other studies on CPZ models in related fields regarding the upregulation of SQSTM1/p62 at elevated levels of autophagy; the reasons for this are unknown at this time [34].

We demonstrated that FKBP5 contributes to autophagy in a subsequent validation of LC3B, and was increased to decrease during the development of the pathological environment (Fig. 7I). This was accompanied by a type I to II transition, which was markedly lagging in the FKBP5 mice (Fig. 7J). This again showed a more inefficient recovery in FKBP5^ko^ mice. In view of our findings on the regulatory role of Fkbp5 on mitochondria and the differences between Fkbp5^ko^ mice and WT mice in the trend of LC3B changes, we considered that it may affect mitophagy through the PINK1-Parkin pathway. To verify whether it is also regulated through other pathways, we chose BECN1 as a non-PINK1-Parkin mitophagy pathway for validation of autophagy markers (Fig. 7K), and no differences in BECN1 expression during the onset period were found between mice of different genotypes.

### FKBP5 accelerates mitochondrial proliferation and mitophagy in tissue cells in a demyelinating environment in mice

**Fig. 8:**
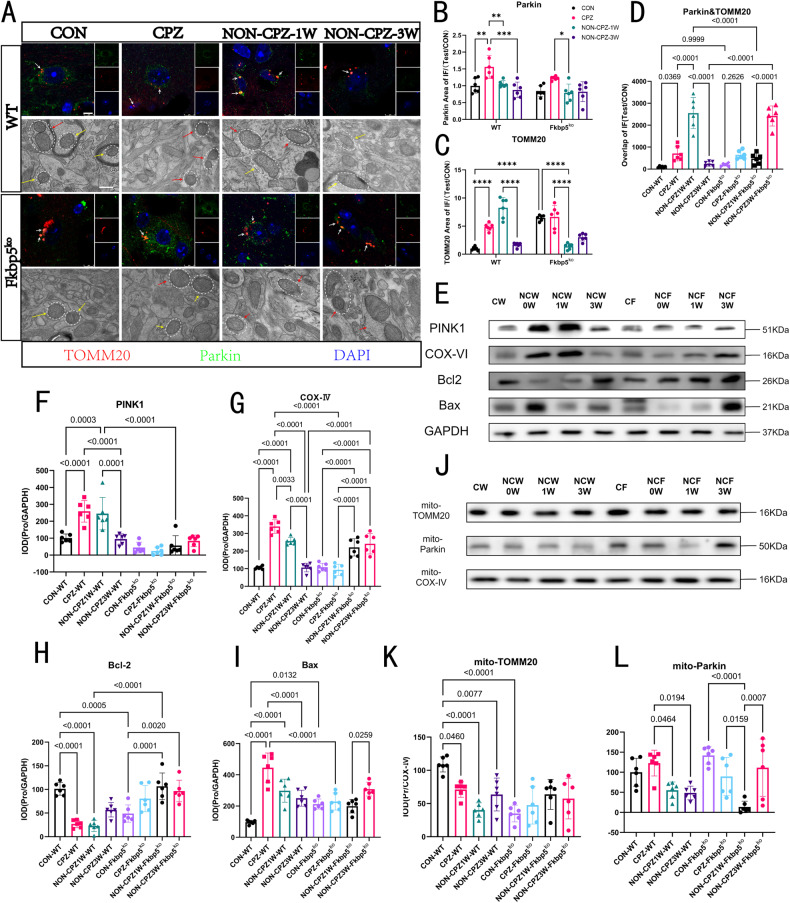
FKBP5 promotes mitophagy. **A** Immunofluorescence images of Parkin and TOMM20 co-staining in the corpus callosum and microscopic confocal images, scale bars = 5 μm, white arrows indicate overlapping locations, suggesting the extent of pathological or mitophagy occurring; transmission electron microscopy mitochondrial views, scale bars = 500 nm, yellow arrows point to normal mitochondria (normal morphology and clear lining), red arrows point to abnormal mitochondria (abnormal morphology and blurred lining). **B**–**D** Fluorescence intensity statistics of both proteins individually and overlapping counts (*n* = 6, analysis method two-way, one-way, respectively). **E** Results of total protein assays for PINK1, COX-IV, Bcl2 and Bax protein blots with internal reference selection of β-Tubulin. **F**–**I** Show the statistics of the corresponding target proteins in the whole-protein WB experiments (*n* = 6, one-way). **J** Extracted mitochondrial proteins and quantitative analysis of the protein blotting results performed for TOMM20 and Parkin. **K**, **L** Mitochondrial protein TOMM20 and Parkin protein blotting statistical results with internal reference selection COX-IV (*n* = 6, one-way).

Similarly, mitophagy occurred mostly in CPZ-treated oligodendrocytes (Supplementary Fig. S3B). Various stimuli that cause MS-like demyelination affect mitochondrial function and reduce mitochondrial mass, leading to increased autophagy and mitophagy. The increase in autophagy may be viewed as an attempt to provide new metabolic intermediates to compensate for the energy deficit, and mitophagy may aim to reshape the mitochondrial population, eliminating damaged mitochondria [35]. To verify the effect of FKBP5 on the mitochondria, we first examined the coloration of TOMM20 and Parkin (Fig. 8A and Supplementary Fig. S4B) and specifically found that mitophagy was active in WT mice, which mainly manifested at zero weeks to one week of recovery, whereas FKBP5 mice reached a similar level only at three weeks of recovery (Fig. 8B–D). To observe the changes in mitochondrial morphology more intuitively, we obtained morphological images of each group separately by TEM (Fig. 8A) and found that the mitochondrial structure of WT mice in the CON group was normal, with a clear and intact inner membrane, and the cristae were aligned in parallel. However, the number of mitochondria increased markedly with CPZ modeling, with curved fluctuation of the inner membrane, and an increase in the number of suspected mitochondrial autophagosomes, which increased with gradual recovery and improvement. However, this phenomenon was not obvious in the FKBP5 group until three weeks after.

To further verify the dynamic changes in mitophagy, we used immunoprotein blotting to examine PINK1, COX-IV, Bcl2, Bax, Parkin, and TOMM20 in total protein (Fig. 8E–I, and Supplementary Fig. S4C) and Parkin and TOMM20 in mitochondrial proteins (Fig. 8J). To demonstrate the efficiency of mitochondrial protein extraction, COX-IV expression levels of total protein, mitochondrial protein and non-mitochondrial protein were examined separately (Supplementary Fig. S1A). We found that both PINK1 (Fig. 8F) and Bcl2 (Fig. 8H) could effectively demonstrate that the increase in FKBP5 promotes mitophagy. The development of the pathological environment is accompanied by the proliferation of mitochondria, in which FKBP5 plays a regulatory role, similar to that of a brake pad when a car is moving too fast. Furthermore, in the quantitative analysis of mitochondrial proteins, TOMM20 (Fig. 8K) and Parkin (Fig. 8L), showed that this regulation can significantly affect mitochondria, and Parkin was inhibited during the recovery period in FKBP5 mice.

### PPAR-γ can affect mitophagy by acting on PINK1

**Fig. 9:**
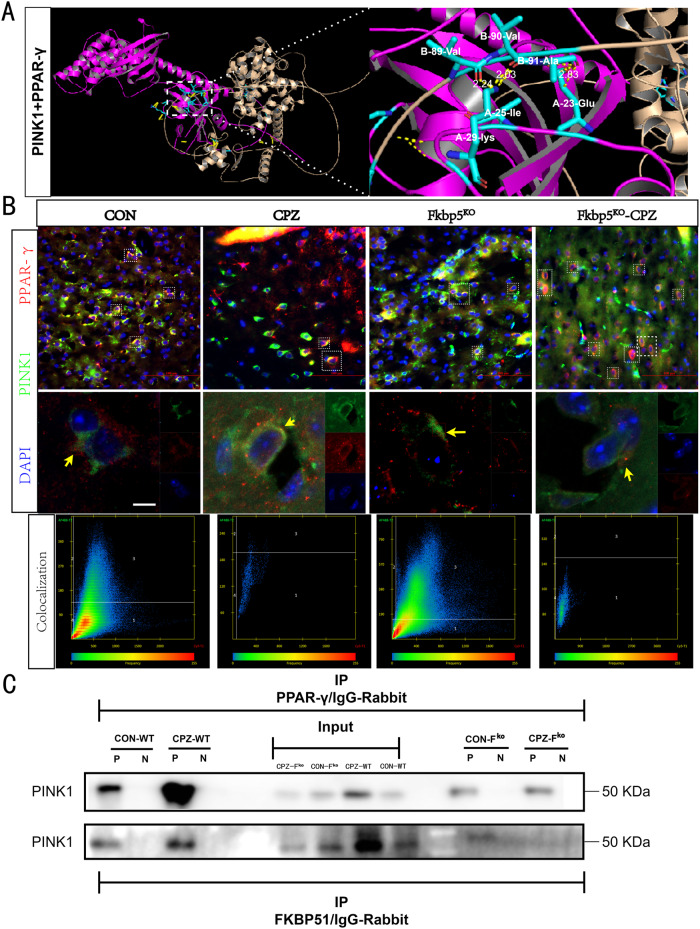
Compared to FKBP51, PPAR-γ possesses a stronger direct interaction with PINK1. **A** Visualization part of the rigid molecular simulation of docking between FKBP51 and PPAR-γ, with a free energy level of −3.3 to −3.5 kcal/mol, spontaneous docking is considered possible. **B** Fluorescence results of PPAR-γ co-staining with PINK1, scale bars = 50 μm, obtained by confocal microscopy and statistics from boxed area colocalization analysis, scale bars = 5 μm, are shown separately. **C** The results of docking with mouse-derived FKBP51 antibody and CoIP results obtained with rabbit-derived PPAR-γ antibody, indicating an interaction among these three proteins. The input results indicate non-IP WB bands, both proteins are detectable and negative control results (N) with weak or no signal, and a weaker signal for PINK1 was obtained with the FKBP51 antibody.

After above analyzing the molecular trends of PPAR-γ at the RNA and protein levels (Fig. 6G, H) and PINK1 at the protein level (Fig. 8E, F), we found that there was indeed an antagonistic relationship between the two classes of proteins, but there was no evidence of a direct regulatory relationship between the two. Therefore, we first performed molecular simulations of docking between PPAR-γ and PINK1 (Fig. 9A). Subcellular localization showed that the PPAR-γ-enriched region also contained the cytoplasm, whereas PINK1 was enriched in the outer mitochondrial membrane, where conditions for direct contact exist (Supplementary Table S1). Subsequently, we obtained immunofluorescence staining images of the two proteins and clearly found that the expression of PPAR-γ was decreased in WT mice after CPZ modeling, whereas the expression level was up-regulated in Fkbp5^ko^ mice, suggesting that stress up-regulation of PPAR-γ is difficult to inhibit in a state of diminished FKBP5 regulation (Fig. 9B). Subsequently, the colocalization levels of the two proteins were analyzed using the built-in software of the confocal microscope (Fig. 9B), which showed Pearson coefficients above 50% and Manders coefficients above 70%, suggesting a high degree of direct correlation between the two classes of proteins. CoIP was performed to target PPAR-γ, FKBP51, and PINK1 (Fig. 9C). PPAR-γ interacted with PINK1, while the interaction between FKBP51 and PINK1 was weaker, suggesting that FKBP51 indirectly regulates PINK1 via the PPAR-γ pathway.

## Discussion

FKBP5, a newly identified protein with abnormal expression in MS progression, accumulates in human MS [29, 30]; however, the physiological role and mechanism of FKBP5 in CPZ pathogenesis remains unclear. In this study, we found that FKBP5 is crucial for the progression of myelin loss and regeneration by activating the PINK1/Parkin-mediated mitophagy pathway, in which the important regulator, PPAR-γ, is critically regulated by FKBP5. Additionally, the progression of myelin loss and regeneration in FKBP5-deficient mice treated with CPZ for the same duration was significantly slower than that in WT mice, and mitophagy played an important regulatory role in this process.

Demyelinating diseases, including MS, are characterized by recurrent episodes and progressive neurological deterioration in patients [36]. MS is an inflammatory demyelinating disease mediated by T cells [37]. It mainly affects the white matter of the CNS and is characterized by multiple inflammatory demyelination, axonal injury, and degenerative changes [38, 39]. In recent years, in addition to immunomodulatory therapy [4], promoting myelin regeneration has become an important measure [40]. The CPZ-induced demyelination model provides a relatively controlled process, from myelin sheath loss to myelin regeneration. By regulating the time required for CPZ administration, myelin could be restored in animals after acute demyelination. Therefore, this model is suitable for studying the mechanisms of myelin loss and regeneration [41]. In this study, CPZ was administered to mice for six weeks, the decline in movement ability and brain tissue, especially the corpus callosum, was accompanied by demyelination lesions in WT mice (Fig. 1C, E, F). Notably, the degree of myelin loss in Fkbp5^ko^ mice was significantly lower than that in WT mice under the same CPZ induction conditions. We designed one-week and three-week recovery groups based on the mice model (Fig. 2A) and compared the differences in trends between WT and Fkbp5^ko^ mice under this dynamic process. The absence of FKBP5 lead to severe decrease of the pathological demyelination environment process (Fig. 2C–F), suggesting a role for FKBP5 in lesion myelin loss. Additionally, FKBP5 may also be involved in promoting myelin recovery or improving the myelin environment.

Mitochondria are the main organelles providing energy to the body [42]. Mitochondrial dysfunction is closely associated with many diseases, including functional metabolism and neurodegenerative diseases [23, 42]. Oligodendrocytes are known to be particularly susceptible to pathologic events, such as hypoxia, excitotoxicity, reactive oxygen, and nitrogen species, and the subsequent loss of oligodendrocytes has been functionally linked to mitochondrial dysfunctions [43]. CPZ consistently induces the abnormal production of large mitochondria in hepatocytes and oligodendrocytes [44]. These very large mitochondria are caused by a lack of cytochrome C oxidase in the oxidative phosphorylation process, caused by the reduction of copper ions in the mitochondria, affecting mitochondrial respiratory function and ultimately causing mitochondrial morphological changes and metabolic damage [45, 46]. In addition to decreased cytochrome C oxidase activity in the mitochondria of oligodendrocytes of the callosum after feeding CPZ to C57BL/6 N mice, the activities of copper and zinc superoxide radicals significantly decreased, indicating that CPZ affects the mitochondrial function of oligodendrocytes and leads to their apoptosis, eventually resulting in myelin detachment [46, 47]. Consistent with these findings, the mitochondria in the corpus callosum of the CON group had elliptical structures with normal ridge morphologies and a more abundant endoplasmic reticulum on the brown side, whereas the mitochondria in the CPZ group showed variable morphologies and increased mitochondrial autophagosomes. However, the mitochondria in the Fkbp5^ko^ group had more abnormal morphologies (Fig. 8A), including more blurred ridges and more rounded and expanded mitochondrial outer mold structures, but the frequency of autophagic mitochondria was relatively low, both of which indicated that Fkbp5^ko^ mice resisted the self-repair mechanism after CPZ-induced mitochondrial damage.

Mitophagy, mediated by the PINK1-Parkin pathway, is the most extensively studied pathway in mammalian systems [25]. Mitochondria are the energy factories of cells and are involved in many important physiological processes such as cell metabolism, signal transduction, differentiation, growth, apoptosis, and death [48–50]. When the mitochondria are damaged, they release pro-apoptotic factors that cause cell death. Mitophagy is the selective removal of dysfunctional mitochondria to achieve a balance between the quantity and quality of mitochondria and the stability of the intracellular environment and is an important mitochondrial protection mechanism [51, 52]. Demyelination is associated with mitochondrial damage [53]. PINK1, a mitochondrial membrane protein, is capable of sensing mitochondrial damage and is recruited to damaged mitochondria to activate the downstream E3 ubiquitin ligase PRKN and ubiquitin, forming a complex that enables damaged mitochondria to be promptly cleared by lysosomes, thereby protecting nerve cells. Mitochondrial impairment as a feature of MS [54, 55]. Alterations in mitochondrial function and dynamics are associated with autophagy [56]. OCR is a valuable indicator of mitochondrial respiratory capacity and energy production. Therefore, we tested OCR in primary brain tissue cultures and found CPZ induced a decrease in OCR, and that Fkbp5 deletion also significantly inhibited OCR (Fig. 5H). Additionally, we tested the production of ATP after the application of CPZ, and the results were consistent with those of OCR (Fig. 5I). To demonstrate corrupted mitochondrial function, we also verified mitochondrial activity by detecting MMP. CPZ or *FKBP5* deletion lowered MMP (Fig. 4I). Therefore, *FKBP5* deletion alters mitochondrial function.

Our RNA-seq analysis showed that the development of the pathological milieu in CPZ-induced demyelination of Fkbp5^ko^ mice was delayed mainly by the blockage of autophagy and changes in the mitochondrial membrane fraction (Fig. 4). This should be extensively activated in the WT mouse CPZ model, including the Wnt signaling pathway [15], mTOR signaling pathway [57], and GABAergic synapses [58], all of which would be profoundly affected by *FKBP5* gene knockout. The Bcl2 pathway, which is regulated by the estrogen signaling pathway in which FKBP51 is located, also affects the entire autophagic process [59], suggesting that an adequate amount of FKBP5 is essential for activating mitophagy in CPZ-mediated mitochondrial damage (Fig. 4E). Heatmap results showed that FKBP5 deficiency severely impaired the expressions of several genes involved in mitophagy activation, including PINK1, Bag1, SQSTM1, Park2, and TOMM20 (Fig. 4D). PPAR-γ, Bcl2 and Bax are key cytokines that affect subsequent mitochondrial choices, play critical roles in remyelination [60, 61]. FKBP5 deletion lead to a decrease in autophagy levels and mitophagy-related protein expressions (Fig. 4D). FKBP5 also interacts indirectly with PINK1, a major mitophagy protein critically regulated by PPAR-γ (Figs. 6–9). This study was conducted to further investigate how FKBP5 regulates the formation of a benign myelin regeneration environment by directly regulating the expression of downstream signaling molecules for which we now understand that mitophagy plays an important role.

FKBP5 promoted the regulation of autophagy to influence the developmental process of pathological environments by inhibiting PPAR-γ. MS is characterized by neuroinflammation and demyelination with the loss of oligodendrocytes in the CNS [62]. PPAR-γ is an orphan nuclear receptor that is a member of the nuclear superfamily of ligand-activated transcription factors. PPAR-γ expressions are implicated in migration, apoptosis, cell growth, and antioxidant and inflammatory responses [63]. PPAR-γ is normally expressed at low levels in the CNS, but it is also expressed in neurons, oigodendrocytes, astrocytes, microglia/macrophages [64], T and B lymphocytes, dendritic cells [65] and brain endothelial cells [66]. PPAR-γ can repress inflammation by decreasing NF-κB activity [67]. Consistent with these results, we found that in Fkbp5^ko^ mice affected the expressions of PPAR-γ, altering the structure of mitochondria and the level of autophagy in the corpus callosum (Figs. 6, 7). Based on the premise that autophagy activation participate in the regulation of mitochondrial homeostasis and neuroprotection [34, 68], our findings found FKBP5 activates autophagy to eliminate CPZ-induced mitochondrial damage by inhibiting PPAR-γ.

Mitophagy clears depolarized mitochondria and prevents excessive ROS production, which is an important factor involved in cell survival and pathogenesis of MS [69, 70]. Defects in autophagy lead to the accumulation of damaged mitochondria and ROS. Accumulated ROS is critical for demyelination and axonal loss. Autophagy clears damaged mitochondria to reduce excessive ROS production, which is protective against MS [71]. Hypoxia/reoxygenation inhibited the expression of PPAR-γ but increased the expressions of LC3II, Atg5, SQSTM1/P62, and PINK1 in a time-dependent manner [72]. These results showed that PPAR-γ protein expressions were negatively correlated with that of mitophagy. Consistent with these results, FKBP5 promotes the activation of PINK1-mediated mitophagy by inhibiting the activity of PPAR-γ during CPZ-induced demyelination and remyelination (Figs. 6–8). However, due to the high expression of FKBP51 in the pathological state to dissipate PPAR-γ, mitophagy is liberated accordingly. Mmitochondria proliferate to ensure the energy supply for repairing the pathological environment, and excessive mitochondrial work also promotes an excessive burst of ROS (Fig. 5C), which can partly explain the mechanism of inflammation in the pathological environment. As we knocked out FKBP5, the activation of the mitophagy pathway of PPAR-γ-regulated PINK1 was inhibited before the increased amount of FKBP5 was restored to normal values, such that the onset phenotype and recovery time both lagged (Figs. 6–8). Peroxisome proliferator-activated receptor gamma coactivator-1 alpha (PGC-1α/PPARGC1A) is a pivotal transcriptional coactivator involved in the regulation of mitochondrial metabolism, including biogenesis and oxidative metabolism [73]. Therefore, the initiation of PGC-1α activation is the key element in the mitochondrial biogenesis process [73, 74]. In the nervous system, the expression of PGC-1α depends on the region of the brain [75]. PGC-1α is crucial for proper functioning of the variety of cell types across the CNS. CPZ has been shown to cause mitochondrial dysfunction in oligodendrocytes and induce oxidative stress in the corpus callosum [76]. CPZ also inhibits mitochondrial function and gene expression, resulting in perturbations in mitochondrial fission and fusion [77]. However, CPZ diminished the expression of PGC-1α gene and its downstream transcription factors, NRF1, TFAM and MFN2 [78]. Consistent with previous findings, our transcriptome results showed that CPZ reduces PGC-1α expression levels in the corpus callosum, followed by a return to normal levels at three weeks after removal of CPZ. However, PGC-1α did not differ with the deletion of Fkbp5 or the development of pathological environment (Supplementary Fig. S2). This gene may not be involved in the pathway regulated by FKBP5 and we speculate that there may be other regulatory pathways. The process of CNS demyelination and regeneration in an environment where myelin is disrupted is an extremely complex system, and the present findings are one of the many mechanisms that previous studies have pointed out [79]. We demonstrate that FKBP5 affects mitophagy through communication with PPAR-γ-PINK1 and that FKBP5 indirectly affects the PINK1 mitophagy pathway. In brief, the pathological environment requires elevated energy metabolism and consequent massive proliferation of mitochondria. Although mitophagy effectively cleans diseased mitochondria to inhibit ROS production and inflammation, these responses still occur because of an increased number of mitochondria. Mitophagy acts as a buffer to ensure increased inflammation and energy metabolism in the early stages of pathological development and is an indispensable drivers of the development of the pathological environment.

In summary, our study is the first to report the physiological role of FKBP5 as a key mediator of the progression of myelin loss and regeneration during CPZ. We propose a model in which FKBP5 is highly expressed in the environment of abnormal myelin loss and can exert its PPIase activity to eliminate the PPAR-γ-mediated mitochondrial survival mechanism to activate the PINK1-parkin mediated-mitophagy signaling pathway and create a benign myelin regeneration environment. Our study sheds light on a novel regulatory mechanism through which FKBP5 modulates mitophagy and may be a potential target for the diagnosis and treatment of central demyelination-associated diseases.

## Methods

### Animals

FKBP5 knockout C57BL/6 N mice (Fkbp5^ko^), No. EGE-ZQ-047 was provided by the Biocytogen Co., Ltd. (Beijing, China). The *FKBP5* gene is located on mouse chromosome 17 (87.4 kb NCBI ID: 14229). The mice used in this study were knocked out of exon 35 of *FKBP5*, an approximately 10 kb fragment, using the CRISPR/Cas9 method. F0 generation Fkbp5^ko^ mice, identified as heterozygote, were transferred to the laboratory and animal house. WT C57BL/6 N mice (WT) were purchased from Viton Lever Laboratory Animal Technology Co., Ltd. (Beijing, China). WT mice used for the experimental study were littermates of Fkbp5^ko^ heterozygous mice. At the start of the experiment, all male mice were 10–12 weeks and 13–15 weeks of age, corresponding to mice undergoing a three-week recovery period. The investigator was blinded to the group allocation of animals during the experiment.

### CPZ-induced demyelination mice model

A model of acute demyelinating disease was established by feeding 0.2% CPZ to WT mice and FKBP5 heterozygous mice for six weeks to simulate different degrees of spontaneous myelin regeneration processes. It was demonstrated that the same type of mice in this model could return to a near-normal state after a three-week recovery period. The success of this model was assessed through behavioral experiments using rotarod test.

### Rotarod test

The rotarod test was used to assess the movement abilities of the mice. Before the experiment, all mice were behaviorally trained to move on the rotating bar at 10 rpm. When they grasped the rotating bar or the bar fell and stopped moving, the animals were removed and retrained for 3 min. Mice that exercised at 10 rpm for 3 min were included in this study. At the end of the training, the mice in the different treatment groups were formally evaluated for movement ability by moving them at 30 rpm on a baton spinner, and the time the mice spent moving on the baton were recorded. The longer the exercise time, the better was the movement ability of the mice. The diameter and length of the batons used in this experiment were 30 and 60 mm, respectively, and five mice were evaluated simultaneously in each experiment.

### Cell culture and simulated inflammation

Mouse BV2 microglia were purchased from Yubo Biologicals, and all cells used for the experiments were passaged for to 4–7 generations to ensure that subsequent experiments were not affected by multiple passages. The same number of passages was maintained between cells from the same batch. Cells were maintained in high-sugar Dulbecco’s modified Eagle’s medium (DMEM) supplemented with 10% fetal bovine serum, without 1% penicillin and streptomycin, to prevent antibiotic interference, which did not affect the overall cell growth based on observations during one week of preculture. The cells were maintained at 37 °C in a humidified environment with 5% CO_2_, 95% air. BV2 cells were incubated with 10 μg/mL LPS in humidified 5% CO2, 95% air for 6 or 24 h at 37 °C. It has been documented that LPS induction of BV2 can predominantly produce TNF-α [80].

### Establishment of FKBP5 overexpressing BV2 cell line

Plasmid design and lentivirus packaging were performed by Obio Technology (Shanghai, China), and the titer of the virus stock solution was tested at 1.0 × 10^8^ UI stored at −80 °C. According to the pre-experiment, 20 MOI was used and 500 μL of the BV2 cell line was infected by preheating in advance according to the percentage of virus stock solution of 0.4%. The conditions were used for incubation for 14–16 h and then passaged and cultured for subsequent experiments.

### Transfection (RNAi)

This experiment was designed for 24- and 96-well plate cell cultures; the reagents used for transfection, Lipo2000 (GENERAL BIOL) and siRNA (GenePharma) were at the same concentrations. Volumes of 1 and 10.25 μL of Lipo2000 were added to each well for both plates, respectively, and the concentration of the stock solution was 20 μM of siRNA. For the configuration process, DMEM without serum was added in amounts of 100 μL and 20 μL, respectively. The solutions were mixed thoroughly and left for 15 min, and mixed at a ratio of 1:4 with complete medium to form the working solution. Finally, 500 μL and 100 μL of working solution were added to each well of the two plates. The plates were incubated for 6–12 h to change the solution. Incubation continued for 48–72 h to ensure that the RNAi process was completed and used for subsequent experiments. Silencing involved targeting two genes, *Fkbp5* and *Pparg*.

The sequences of the siRNAs were as follows:

*Fkbp5*-siRNA:

forward′5-CAGGCGUUAUCCGUAGAAUTT-3′,

reverse′5-AUUCUACGGAUAACGCCUGTT-3′.

*Pparg*-siRNA:

forward′5-CCGUAGAAGCCGUGCAAGATT-3′,

reverse′5-UCUUGCACGGCUUCUACGGTT-3′.

### ROS and red-fluorescent labeled active mitochondria assay

The cells were divided into four groups: CON, Si-FKBP5, Si-PPAR-γ, Lv-sh-FKBP5-Si-PPAR-γ, meanwhile, blank control and LPS induction were set up in each group to reflect the regulatory differences of target genes in the pathological environment. After this, we gave cells a 30 min Rosup ROS induction mixture treatment. We uesd a Bain-Marie ROS kit, and the cells were inoculated in 96-well black plates constructed of environmentally friendly materials. Fourteen groups were treated in parallel for each group. After the above treatment was completed, the solution was changed to serum-free MEM medium, and 0.2 μL of DHFH-DA (10 mM) and 0.2 μL of mitochondrial red fluorescent probe working solution (Bain-marie; 50 μL of master mix with 420 μL DMSO were used to make the working solution) were added to each well and incubated for 30 min in the dark. The fluorescence intensities were observed with an automatic enzyme marker after the solution change, where ROS was at 488–525 nm and 579–599 nm were active mitochondria. Another cell crawl was performed at the same concentration, stained, and sealed with DAPI, and morphological images were obtained using a laser confocal microscope.

### Brain tissue preparation

Mice were anesthetized with 1% pentobarbital and perfused transcardially with 4% cold paraformaldehyde (PFA) in 0.1 M PB after an initial flush with 0.01 M phosphate-buffered saline (PBS). Brains were postfixed in 4% PFA in 0.1 M PB at 4 °C for 24 h, followed by dehydration in 30% sucrose in 0.01 M PBS until submerged. Brains were embedded in optimal cutting temperature compound (Leica, CM1950, Germany) and cut into 10–20 μm-thick coronal sections using a cryostat microtome (Cryostar NX50, Thermo Fisher, USA). A 20-μm section was used for global staining of the corpus callosum region to observe the overall myelin loss status. A 10-μm section was used for local observations to evaluate protein interactions. The slices were kept in a cryoprotected which was mixed with PBS and at 4 °C for storage.

### LFB staining

Frozen sections from each group used for histopathological staining were pre-warmed at indoor temperature, placed in xylene I and II for 30 min, and then placed in graded alcohol solutions of 100%, 100%, 95%, 95%, and 80% for 5 min each. The sections were rinsed twice with distilled water. A series of sections were stained with 0.1% LFB (Sigma‒Aldrich) solution, sealed for 8 h at 60 °C, washed with distilled water, and placed in 95% alcohol for 10 min. Each mixture was separated in a 0.05% lithium carbonate aqueous solution (Leagene, Beijing, China) for 10 s, and in a 70% alcohol solution for 20 s. These two steps were repeated until the gray and white matters were clearly observed under a microscope. Sections were dehydrated using a conventional alcohol gradient (80%, 95%, 95%, 100%, and 100% for 2 min), placed in xylene I and II for 10 min, and sealed with neutral gum. Images of the LFB staining were captured using a Nano Zoomer Digital Pathology system (Hamamatsu, Japan). Three blinded experimenters used Image-Pro Plus 6.0 (Media Cybernetics, Rockville, MD, USA) to calculate the staining densities for quantification of the myelin and lesion areas. Five animals were included in each group. For each animal, five spinal cross-sections in the rostral-caudal plane taken from the level of injury were analyzed.

### Immunofluorescence staining (IF) and images acquisition

For IF, the floating sections were blocked with 5% bovine serum albumin (BSA) and 0.5% Triton-X 100 for 1 h at room temperature, followed by sequential incubation with primary antibodies overnight at 4 °C and fluorescent-dye-conjugated secondary antibodies for 1 h at room temperature. The primary antibodies included rabbit anti-MBP [1:200, Abcam, Cat: AB216590); rabbit anti-Iba1/AIF-1 [1:200, CST, Cat: 17198); mouse anti-GFAP [1:200, CST, Cat: 3670); mouse anti-PINK1 [1:100, Abcam, Cat: AB186303); rabbit anti-LC3B [1:100, Abcam, Cat: AB192890); mouse anti-P62/Sqstm1 [1:200, CST, Cat: 88588); mouse anti-FKBP51 [1:50, Abcam, Cat: AB79844); rabbit anti-FKBP51 [1:100, CST, Cat: 8245); rabbit anti-TOMM20 [1:100, Abcam, Cat: AB186735); mouse anti-Parkin [1:100, Abcam, Cat: AB77924); and rabbit anti-PPAR-γ [1:100, CST, Cat: 81B8). The Appropriate Alexa Fluor-conjugated secondary antibodies included donkey anti-mouse, donkey anti-rabbit, and goat anti-rat antibodies [1:1000, Life Technologies). Nuclei were counterstained with DAPI at room temperature. Fluorescent images were captured using a confocal laser-scanning microscope (Olympus, FV 3000, Shinjuku, Tokyo) or a fluorescence microscope (Zeiss, M2, Germany) with excitation wavelengths appropriate for Alexa Fluor (350–380 nm, 488 nm, 594 nm, 647 nm), Cy3 (570 nm), or DAPI (380 nm). A mitochondrial protein extraction A tissue mitochondrial extraction kit (Beyotime, B3606) was used with fresh mouse brain tissue preserved on ice. Tissues preserved in the frozen state were not used. A small pieces of brain tissue, weighing approximately 60 mg, were cut from the corpus callosum. The tissues were washed three times with PBS. This tissue was placed in a centrifuge tube or Petri dish on ice and cut into very fine pieces using scissors or a razor blade. Ten volumes of precooled mitochondrial isolation reagent A or mitochondrial isolation reagent A with phenylmethylsulfonyl fluoride was added prior to use and homogenized in an ice bath approximately 10 times. Note: If the weight of the tissue was 80 mg, it can be assumed that the volume of the tissue is roughly 80 µL, and 800 µL of Mitochondrial Isolation Reagent A should be added. The homogenate was centrifuged at 600 × g at 4 °C for 5 min to obtain mitochondria.

### Superoxide assay

Fresh mice brain tissue was rapidly obtained in a cryogenic environment from the corpus callosum region. Each group was weighed to obtain an equal volume of tissue immersed in PBS buffer prewarmed to 37 °C. The block was sheared into tissue fragments with forceps, and the precipitate was rapidly centrifuged and transferred to trypsin (Thermo Fisher, R001100) for digestion at a 37 °C ambient temperature for 3 min with continuous shaking. Subsequently, the supernatant centrifuged for 30 s. After obtaining the cell precipitate, 200 μL of Superoxide Assay working solution (Beyotime, S0060) was added, and incubated at 37 °C for 3 min. The cell suspension was aspirated and placed in a 96-well plate for dual-wavelength determination, using 450 and 600 nm as reference wavelengths.

### Reverse transcriptase-polymerase chain reaction (RT-PCR)

Total RNA was extracted using TRIzol reagent (Invitrogen) and reverse-transcribed into cDNA using the Prime Script RT Master Mix Kit (TaKaRa). The SYBR Premix and specific primers were used in the Super Script One-Sep RT-PCR system (TaKaRa). The relative expression levels of Nrf2, PINK1, PPAR-γ and FKBP5 with GAPDH were determined by quantitative PCR. RT‒PCR system (Applied Biosystems) and quantitative PCR were performed according to the manufacturer’s instructions. PCR amplification was performed in triplicate. Data were analyzed by 2-ΔΔCt.

Nrf2 forward primer′5-CTTTAGTCAGCGACAGAAGGAC-3′,

reverse primer′5-AGGCATCTTGTTTGGGAATGTG-3′.

Pink1 forward primer′5-TTCTTCCGCCAGTCGGTAG-3′,

reverse primer′5-CTGCTTCTCCTCGATCAGCC-3′.

Pparg forward primer′5-GGAAGACCACTCGCATTCCTT-3′,

reverse primer′5-GTAATCAGCAACCATTGGGTCA-3′.

Fkbp5 forward primer′5-GATGAGGGCACCAGTAACAATG-3′,

reverse primer′5-CAACATCCCTTTGTAGTAGTGGACAT-3′.

Gapdh forward primer′5-AGGTCGGTGTGAACGGATTTG-3′,

reverse primer′5-GGGGTCGTTGATGGCAACA-3′.

### Western bolt

Four- and five-month-old FKBP5^ko^ mice and age-matched WT mice were sacrificed, and brain tissues were removed. The corpus callosum region was isolated and lysed using a rapid cell tissue lysis solution (RIPA) (Beyotime, Cat: P0013B). Protein concentrations were determined using the BCA assay kit (Beyotime, Cat: P0010S). Eight micrograms of protein were mixed with sample loading buffer (Beyotime, Cat: P0015F) and heated at 95 °C for 5 min. Subsequently, the gels were subjected to electrophoresis using 10% and 12.5% sodium dodecyl sulfate-polyacrylamide (SDS-PAGE) gels of 1.5 mm thickness. The gels separated proteins with sedimentation coefficients below and above 70. The proteins were then transferred onto polyvinylidene difluoride membranes (Millipore, Cat: ISEQ0010) in transfer buffer containing 15% methanol in an ice bath at 0.22 A for 50 min. After transfer, the membrane was blocked in 5–10% BSA (dissolved in Tris-buffered saline containing 0.1% Tween-20) for 1–1.5 h. The membranes were then incubated with antibodies at 4 °C overnight. After washing, the membranes were incubated with horseradish peroxidase-labeled secondary antibody (goat anti-mouse IgG, Beyotime, Cat: A0216) or goat anti-rabbit IgG, Servicebio, Cat: GB23303) at a dilution of 1:1000 for 1 h at 37 °C. In addition to the duplicate antibodies mentioned in the previous section, other indicator antibodies included rabbit anti-Nrf2 [1:1000, Abcam, Cat: AB92946); rabbit anti-LC3B [1:1000, Abcam, Cat: AB192890); rabbit anti-Benc1/Beclin1 [1:1000, Servicebio, Cat: GB112053); mouse anti-COX-IV [1:1000, Servicebio, Cat: GB12250); rabbit anti-Bcl2 [1:1000, Abcam, Cat: AB182858); and mouse anti-Bax [1:1000, CST, Cat: 89477). The membranes were then treated with enhanced chemiluminescence substrate (Amercham, RPN2231V1) and visualized using a ChampChemi gel imaging system (Sage Creation, Beijing, China). After stripping with stripping buffer (Beyotime, P0025S) for 40 min, the membranes were incubated with mouse anti-GAPDH [1:5000, Servicebio, Cat: GB15002) or rabbit anti-β-tubulin [1:5000, Abcam, Cat: AB6046) as an internal reference at 4 °C overnight and then incubated with the corresponding HRP secondary antibody at room temperature for 1 h. Protein levels were quantified using Image J 1.53t with GAPDH or β-Tubulin as the standard.

### Primary brain tissue cultures

Rat pups were taken within two days of birth, anesthetized by sinking ice, submerged in 70% ethanol solution for disinfection and execution, and then cleaned with Hank’s Balanced Salt Solution (HBSS) for surface alcohol; the brain was isolated and the cortex was peeled off; the vascular membranes were removed by operation under a dissecting microscope. The processed cortical tissues were washed clean in HBSS, and then clipped into fine tissue pieces using forceps; and the collected tissue pieces were quickly added to the DNase I stock solution (0.2 mg/mL). Trypsin (0.25%) in HBSS was used for several times to homogenize and digest the healthy glial cells for approximately 15 min in total, and then DMEM medium containing fetal bovine serum was added to terminate the digestion. The supernatant was added to the dry culture dish treated with polylysine beforehand, and left at 5% CO2 concentration and 37 °C.

### Flow cytometry

After treating the cells separately according to the pre-designed grouping, the cells were digested with trypsin and washed twice with PBS or HBSS for the assay, which involved two assays: ROS using green fluorescent labeling (Beyotime, S0033S) and JC-1 (Invitrogen, M34152), which needed to be detected for the migration of both red and green colors.

### Oxygen consumption rate

Cells were pre-inoculated in transparent bottom black 96-well plates and treated according to the pre-designed cells. On the day of the test, two columns (3–6 groups in parallel) were taken to add Carbonyl cyanide-p-trifluoromethoxyphenylhydrazone (FCCP) and Rotone as the control, and three columns were selected as the blank control. The pure medium, mixed isobaric (Dojindo, E297) dye, and the cells of the untreated group were added. The cells of the other experimental groups were replaced by the mixed isobaric dye medium immediately after blocking the air with a drop of mineral oil in each well, and the data were collected by the fluorescent zymography at the wavelengths of 500–650 nm at intervals of 10 min and statistically analyzed.

### ATP testing

Cells were inoculated in transparent bottom black 96-well plates, and treated in advance according to the pre-designed grouping. They were incubated for 4 h after the application of drugs. The supernatant was aspirated and added with 50 μL detergent (Abcam, ab113849), vigorously and smoothly homogenized for 5 min, then 50 μL Substrate Solution (Abcam, ab113849) was added and homogenized. The solution was left to stand for 10 min, and the readings were taken with the fluorescence zymography Luminescent parameter setting.

### Co-immunoprecipitation (CoIP)

Protein stock solution (22 µL) was added to 4× Protein SDS PAGE Loading Buffer, and the lysate was diluted to 1 mg/mL, denatured at 100 °C for 5 min and then used as an input control (positive control). Another 200 μg of protein lysate supernatant was added to 2 μg of FKBP51/PPAR-γ antibody (double pull) for antigen-antibody binding (flip incubation for 24 h) at 4 °C. Note that a negative control (equal amount of normal rabbit IgG instead of FKBP51 antibody was added to 200 μg of protein lysate supernatant) was used for each sample for the same 24 h incubation period. Fresh Dynabeads were pretreated with the lysate in an ice bath three times every 10 min. Dynabeads were adsorbed using a magnetic stand, and the lysate was blotted dry. The antigen-antibody mixture from the 24 h incubation was added to the pretreated Dynabeads and incubated for 2 h at 4 °C by turning over to obtain the antigen-FKBP51/PPAR-γ antibody-Dynabead mixture. The antigen-FKBP51/PPAR-γ antibody-Dynabeads were adsorbed with a magnetic holder and the other unbound protein lysate was discarded, which was followed by adding 30 μL of 1×Protein SDS-PAGE Loading Buffer to mix the Dynabeads complex and it was denatured at 100 °C for 5 min. The supernatant was removed as the immunoprecipitated output protein sample. FKBP51/PPAR-γ/PINK1 were examined by WB to verify the correlation effect relationships among the three.

### Transmission electron microscope (TEM)

Tissue preparation and analysis after myocardial perfusion on ice (0.1 M phosphate buffer containing 4% paraformaldehyde and 2.5% glutaraldehyde; pH 7.2–7.4), the brains of mice were collected, and small squares with volumes of approximately 1 m were collected in the corpus callosum region and quickly placed in precooled 2.5% glutaraldehyde fixative for electron microscopy (SbjBio, BL-G014) for storage at 4 °C. Follow-up processing and sectioning were conducted at the Kunming Zoological Institute in Yunnan Province. Sections were obtained using TEM (JEM-2100, Japan) for image acquisition of the target region, with the main objects of interest being myelin, mitochondria, and diseased mitochondria.

### Transcriptome sequencing analysis

Brain tissues from the corpus callosum region of mice were obtained and transported on dry ice to Novogene (Beijing, China), where eight animal samples (*n* = 3) were sequenced using a double-end sequencing (paired-end) method based on the Illumina technology sequencing platform. Based on Unigene, we performed intergroup GO and KEGG analyses, narrowed down the study to the FKBP5 pathway based on the raw data obtained, drew a heatmap, and mined the core genes for PPI analysis. Graphs were drawn using R.Studio (version 4.2.2).

### Stereology and quantification

Brain sections from bregma 1.1 mm to bregma −2.70 mm were collected consecutively from adult mice. The corpus callosum and the hippocampus were included in these slices. For sampling in a systematic random manner, to 5–8 slices were taken from each brain section. Every 20th slice was sampled systematically, and the first slice was sampled randomly from the first 20 slices. To quantify fluorescence-positive cells, at least three representative areas (20× or 40×) were randomly obtained from the corpus callosum and radiation regions of each brain slice.

### Rigid molecular simulation docking

This study involves the simulated docking process between three types of proteins, FKBP51 (5bxj) and PPAR-γ (4y29), PPAR-γ (4y29) and PINK1 (5tr5). The PDB format files of the 3D crystal structures of the proteins were obtained from the UniProt protein database and were all based on artificially modified structures obtained using the X-ray technique. The above selection of the three serial numbers is the result of a combination of Å and included amino acid lengths. Subsequent calculations were performed based on the GRAMM-X computer cluster, and both groups of proteins were analyzed by selecting the former protein as the receptor and the latter as the ligand. The simulated data obtained from the calculations were uploaded to PDBePISA for analysis, and the available data were adjusted visually using PyMOL, with the main operations being the addition of hydrogen bonds and labeling of the core amino acid groups.

### Statistical analysis

Data were plotted and statistically analyzed using the GraphPad Prism software (version 9.0). All values are expressed as the mean ± standard error of mean One-way analysis of variance was used for comparisons between groups larger than two, and unpaired *t*-tests were used for comparisons between two groups, with post hoc analyses of the significant differences between the samples in each group using Tukey’s test. All data were presented with a mean *p*-value of less than 0.05 indicating a statistically significant difference between groups. Significance was reported as: *p < 0.05, **p < 0.01, ***p < 0.001, and ****p < 0.0001.

### Supplementary information

A reproducibility checklist

Supplementary Figures and Table

Full length uncropped original western blots

## Data Availability

The data used and/or analyzed in this study are available from the corresponding author upon reasonable request.
