# Supplementary material for: FKBP5 activates mitophagy by ablating PPAR-γ to shape a benign remyelination environment
Source: Cell Death Dis. 2023 Nov 11;14(11):736. doi: 10.1038/s41419-023-06260-7 (PMC10640650; doi:10.1038/s41419-023-06260-7)
Supplement: Supplementary file 2 — Supplementary Figures and Table [file 41419_2023_6260_MOESM2_ESM.docx]

**Supplementary Figure S1** *related to Figure. 4 & 8*


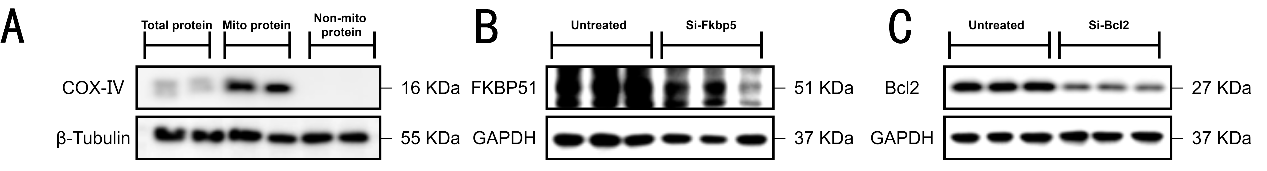


**Supplementary Figure S1** A. To demonstrate the efficiency of mitochondrial protein extraction, COX-IV expression levels of total protein, mitochondrial protein and non-mitochondrial protein were examined separately. B, C. The protein-level siRNA interference efficiencies of primary brain tissue cultures of Fkbp5 and Bcl2. The efficiency of siRNA interference, for each sample, was done in three parallel sets.

**Supplementary Figure S2** *related to Figure. 4*


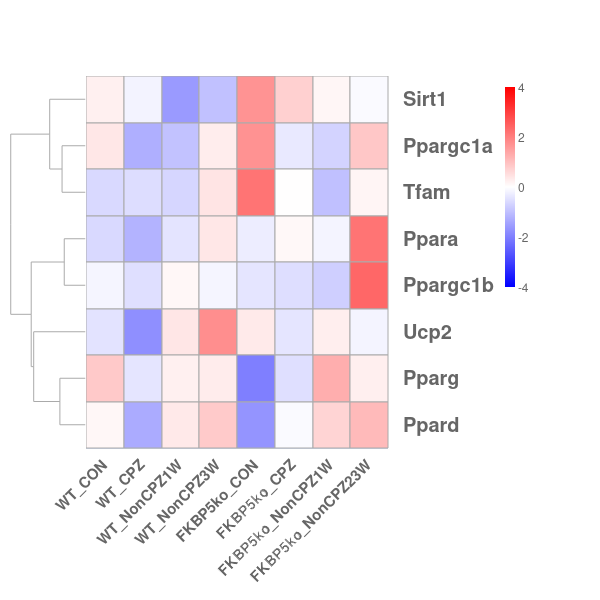


**Supplementary Figure S2** Trends in transcriptome levels of PPAR family-related members at various stages.

**Supplementary Figure S3** *related to Figure. 7 & 8*


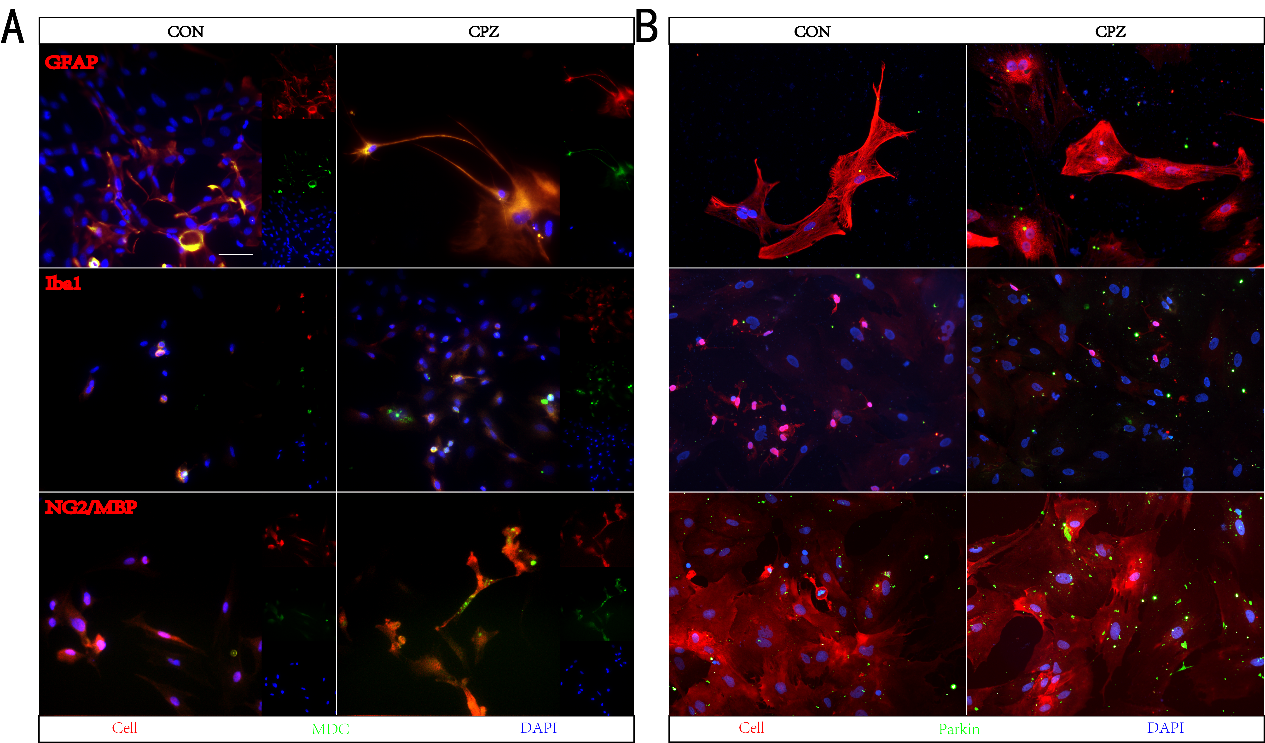


**Supplementary Figure S3** A. Astrocyte marker (GFAP), microglia marker (Iba1), and oligodendrocyte marker (NG2/MBP) were co-stained with autophagy detection reagent, MDC, after applying CPZ-induced treatment to primary brain tissue cultures, respectively. The cell markers were indicated by red light. MDC was excited by green light, and the nuclei of the cells were stained in blue color, Scale bar = 100 μm. B. Cellular markers were selected as in green fluorescence was changed to label the mitophagy marker Parkin. Scale bar = 100 μm.

**Supplementary Figure S4** *related to Figure. 7 & 8*


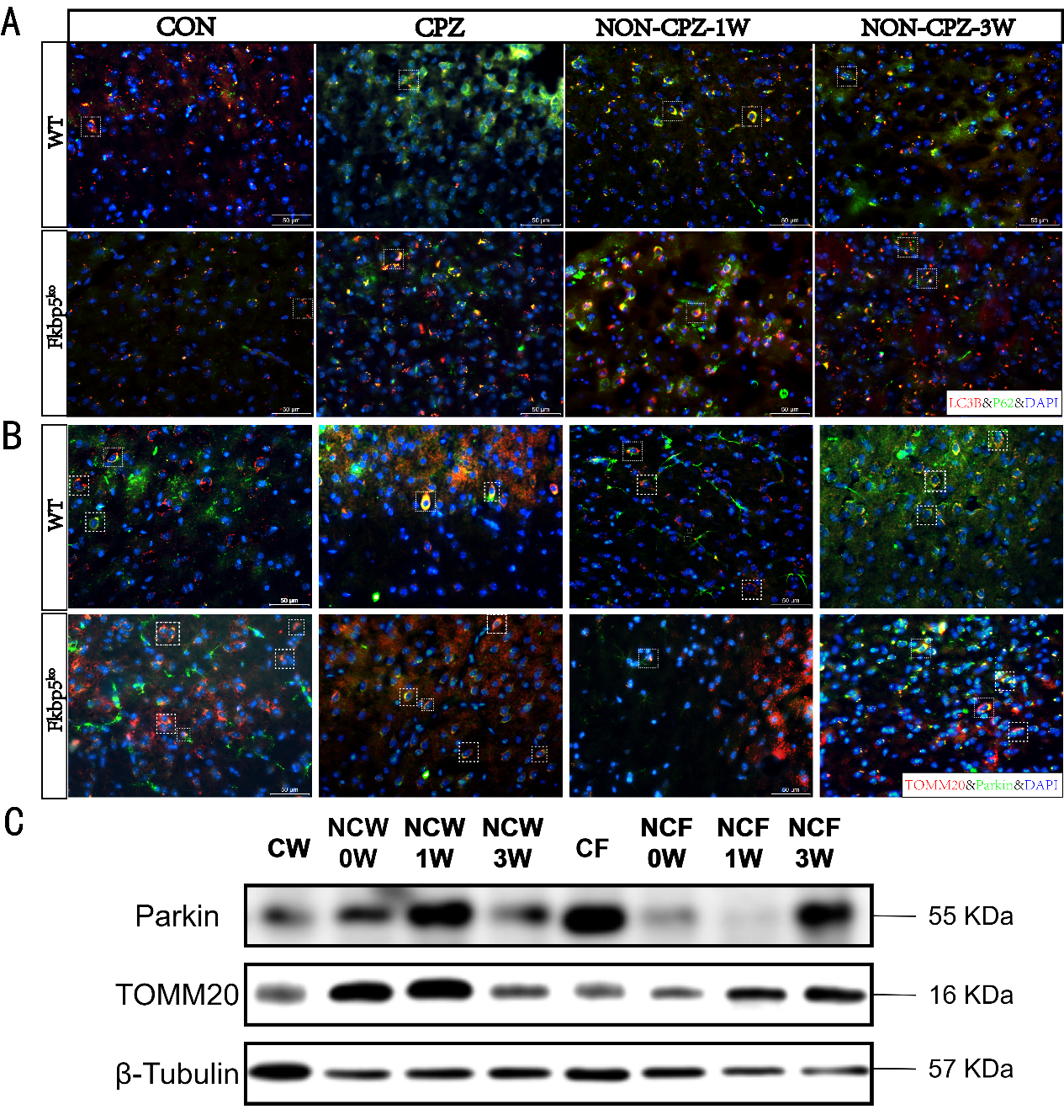


**Supplementary Figure S4** A, B. Low magnification shot fluorograms corresponding to the statistics of Fig. 7.B-D and Fig. 8.B-D, respectively. C. Total protein Parkin versus TOMM20 qualitative protein blotting experiments.

**Supplementary Table S1** *related to Figure. 6*

| id | site | iPSORT | | Amino Acid Content | | | | Misc. |
| --- | --- | --- | --- | --- | --- | --- | --- | --- |
|  |  | [-1_25](https://www.genscript.com/tools/wolf-psort/detail?file=../WoLFPSORTdoc/averageNegativeCharge0_24Description.html) | [MxHy1_30](https://www.genscript.com/tools/wolf-psort/detail?file=../WoLFPSORTdoc/maxHydropathy0_29_12Description.html) | [C](https://www.genscript.com/tools/wolf-psort/detail?file=../WoLFPSORTdoc/CcontDescription.html) | [I](https://www.genscript.com/tools/wolf-psort/detail?file=../WoLFPSORTdoc/IcontDescription.html) | [K](https://www.genscript.com/tools/wolf-psort/detail?file=../WoLFPSORTdoc/KcontDescription.html) | [S](https://www.genscript.com/tools/wolf-psort/detail?file=../WoLFPSORTdoc/ScontDescription.html) | [length](https://www.genscript.com/tools/wolf-psort/detail?file=../WoLFPSORTdoc/lengthDescription.html) |
| FKBP5_HUMAN | cyto_nucl | -6 | -4.2 | 0.02 | 0.048 | 0.116 | 0.048 | 457 |
| PPARg_HUMAN | nucl | -6 | -1.4 | 0.02 | 0.069 | 0.077 | 0.077 | 505 |
| PINK1_HUMAN | mito | 5 | 10.3 | 0.028 | 0.031 | 0.036 | 0.057 | 581 |

**Supplementary Table S1** Subcellular localization data for Fkbp5, Pparg, and PINK1 From the WoLF POSRT website.
