# Supplementary material for: FKBP5 activates mitophagy by ablating PPAR-γ to shape a benign remyelination environment
Source: Cell Death Dis. 2023 Nov 11;14(11):736. doi: 10.1038/s41419-023-06260-7 (PMC10640650; doi:10.1038/s41419-023-06260-7)

# Fig6.B-Fkbp5-IP-FKBP5
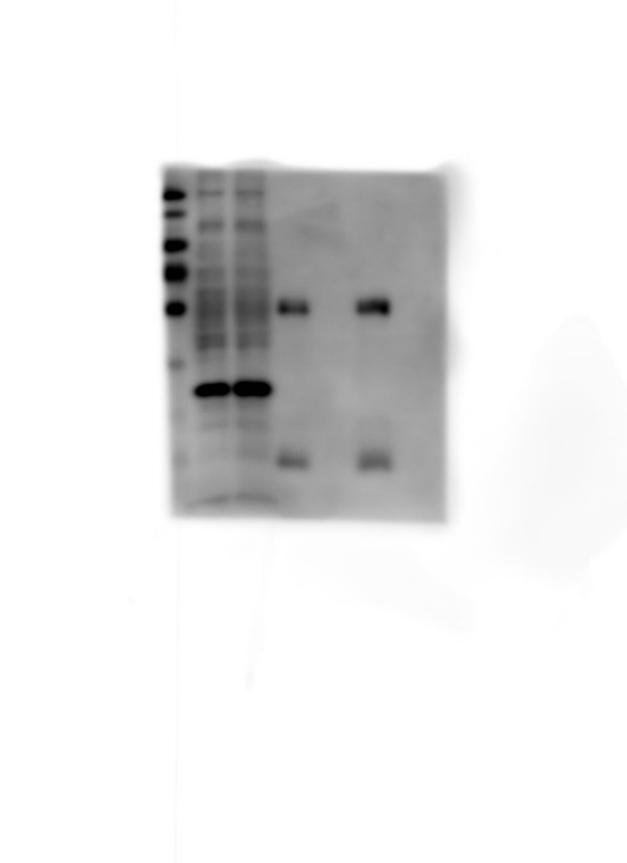

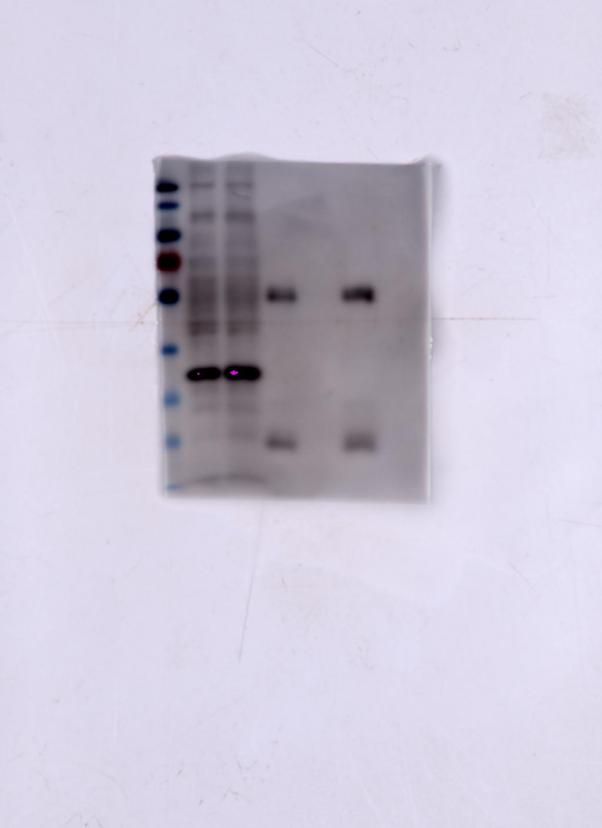


# Fig6.B-Fkbp5-IP-PPAR-γ

#
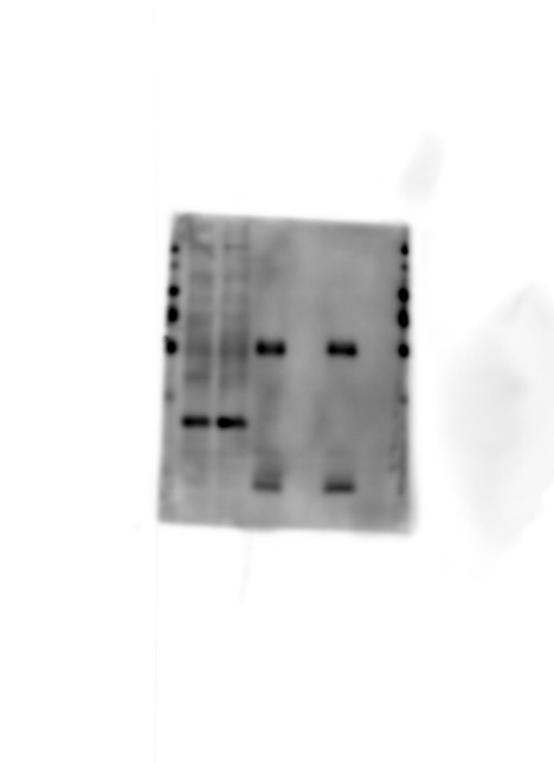

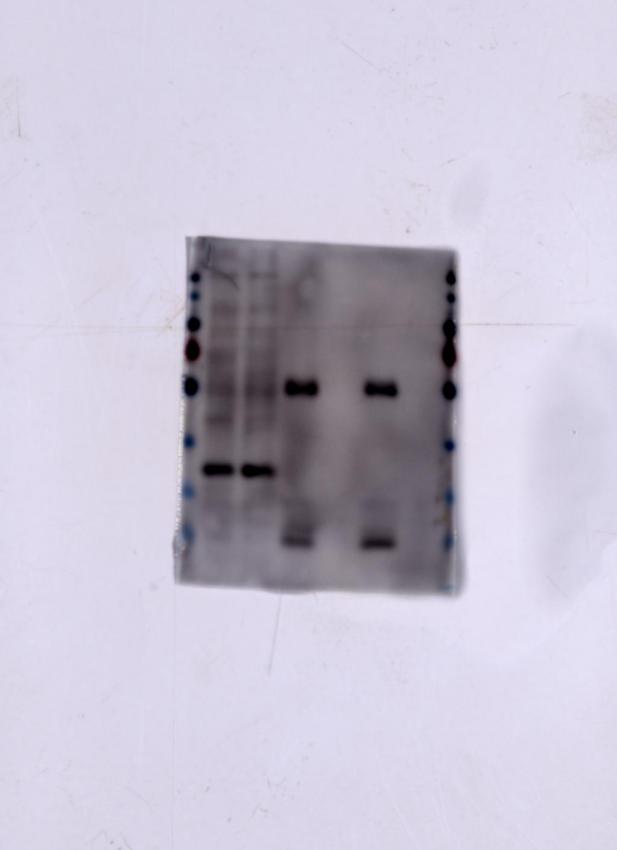


# Fig6.C-FKBP5（up）/PPAR-γ（down）

#
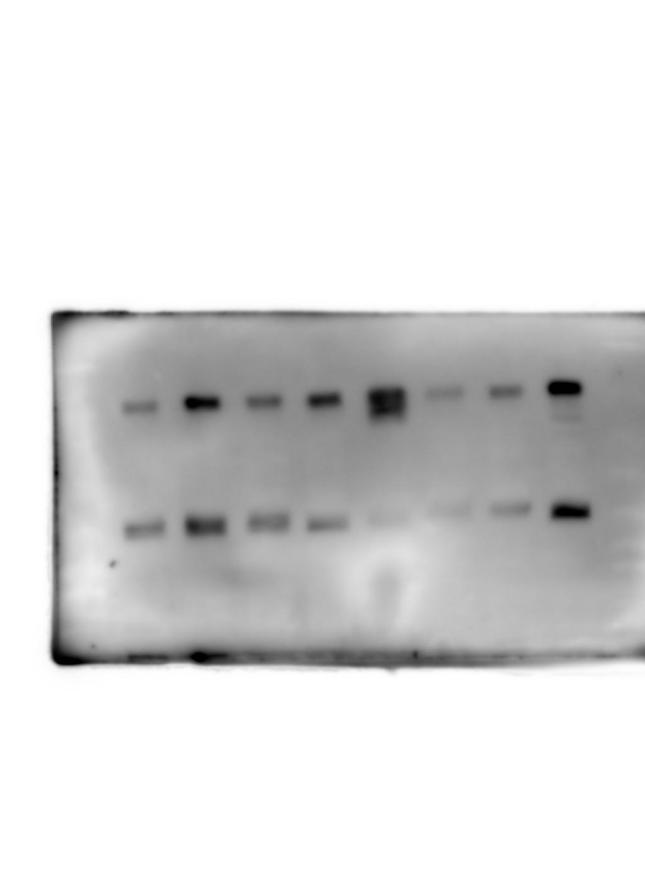

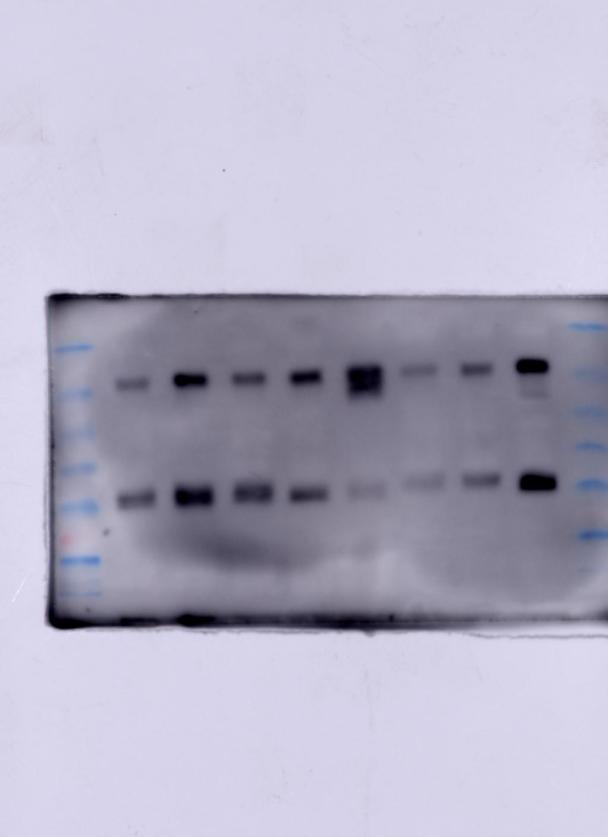

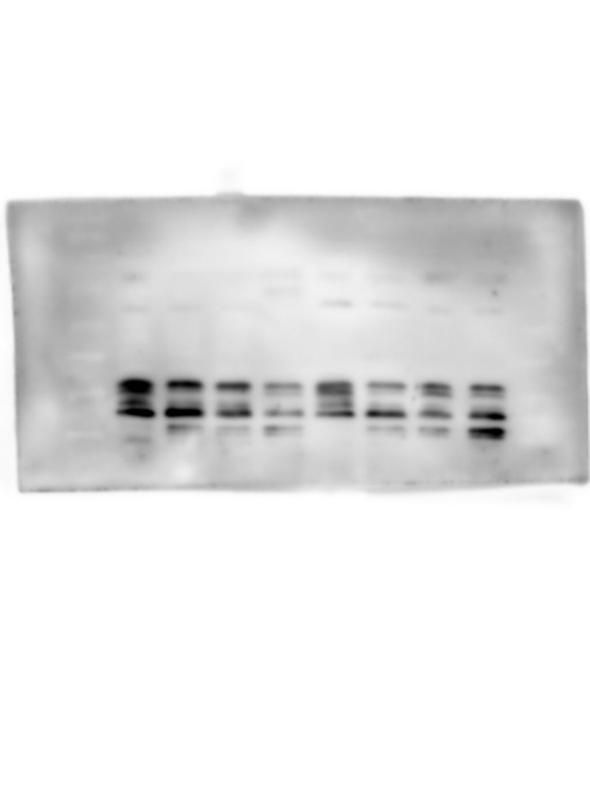

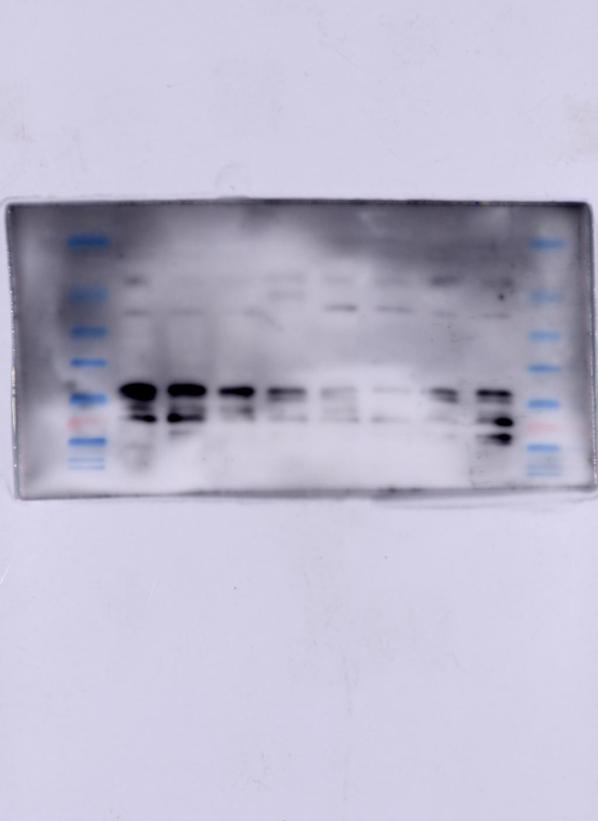


# Fig6.C-β-Tubulin

#
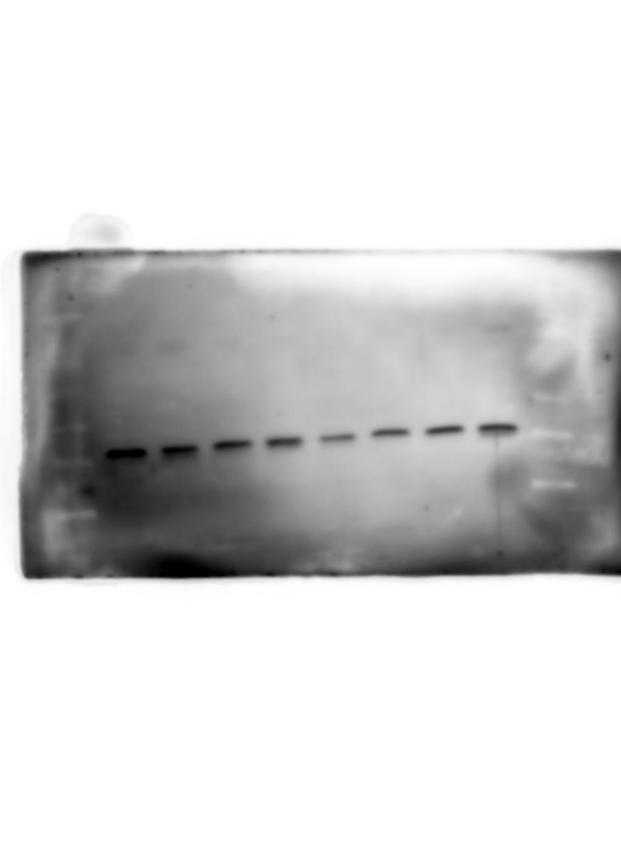

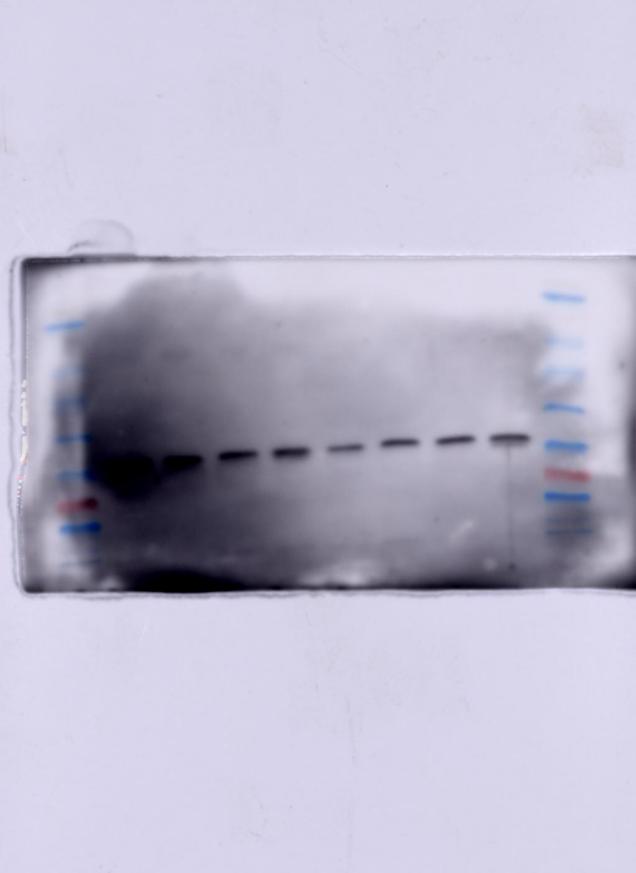


# Fig7.E-LC3B（up）/BECN1（down）


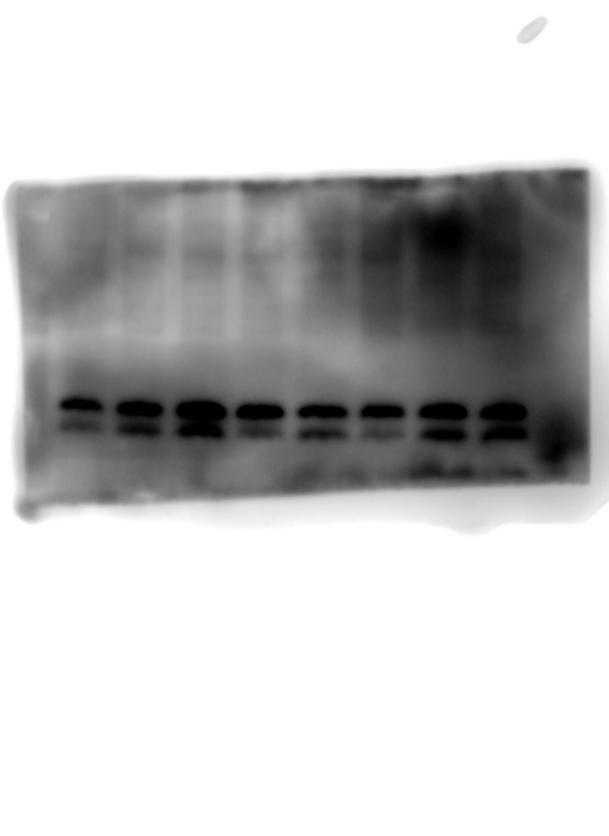

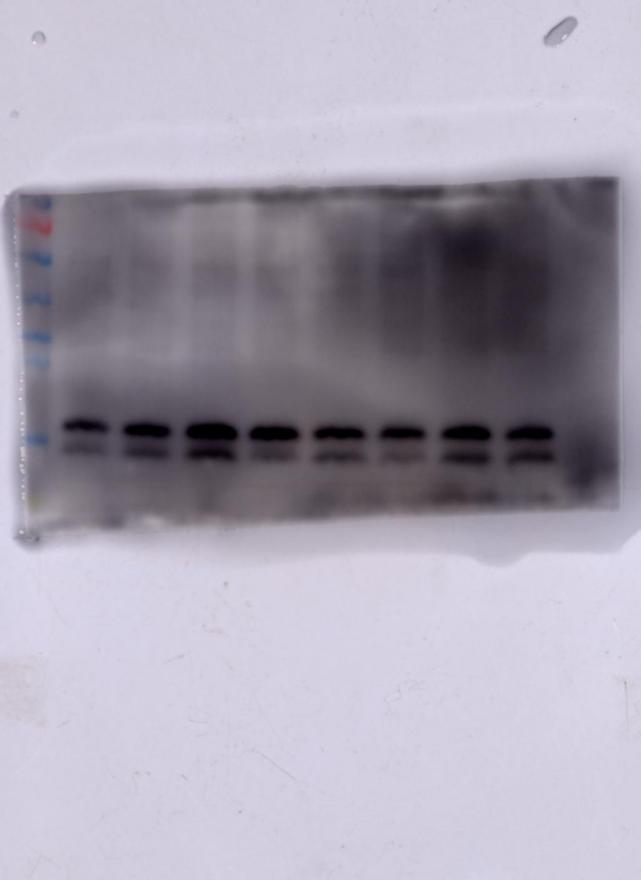


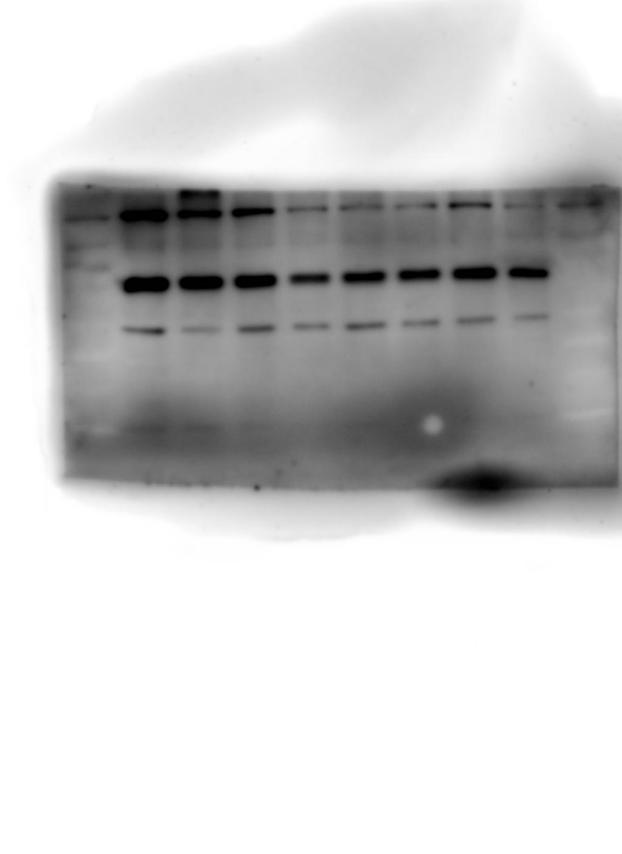

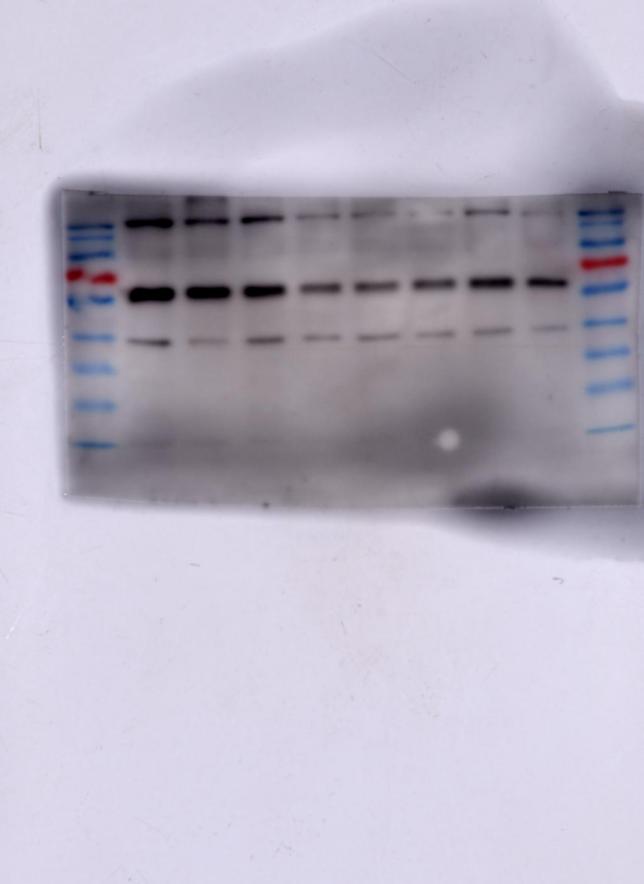


# Fig7.E-Nrf2（up）/p62（down）


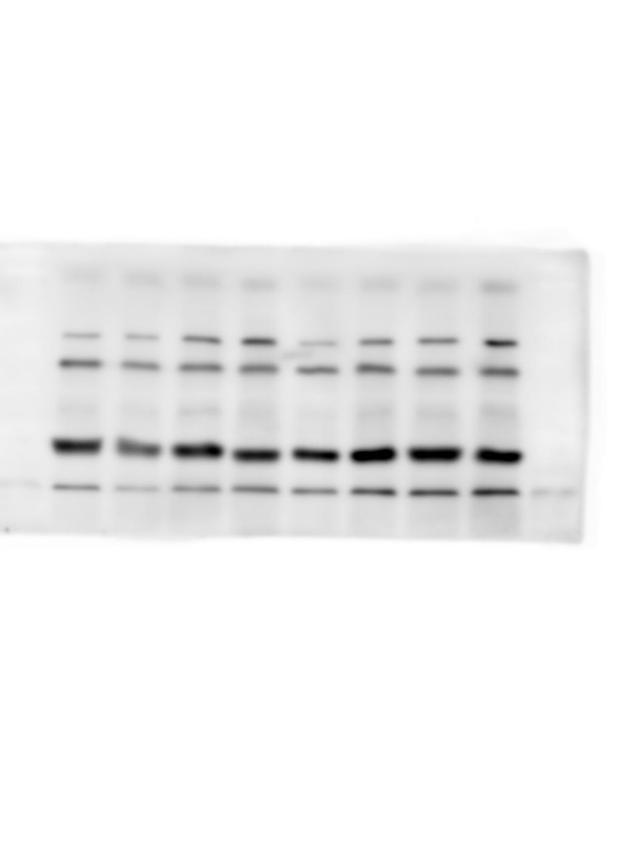

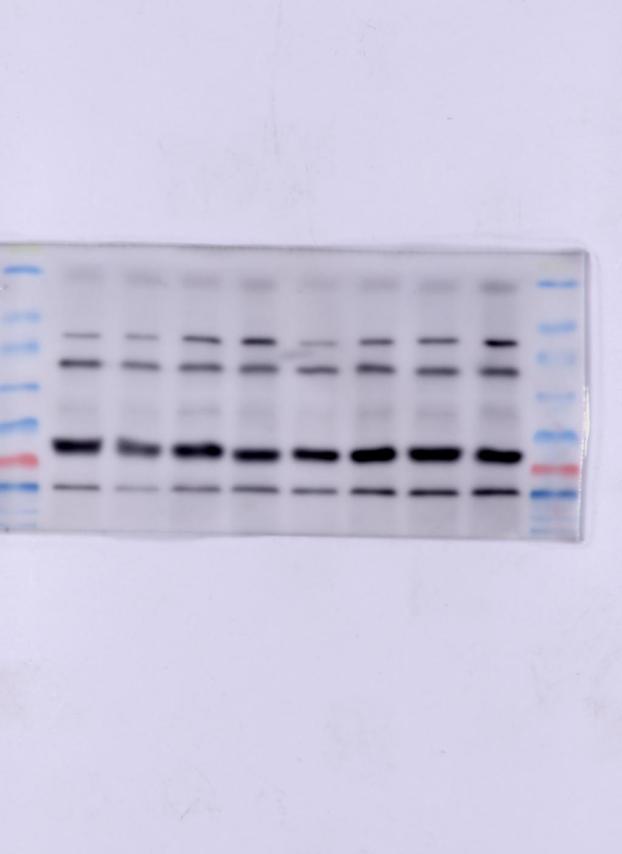


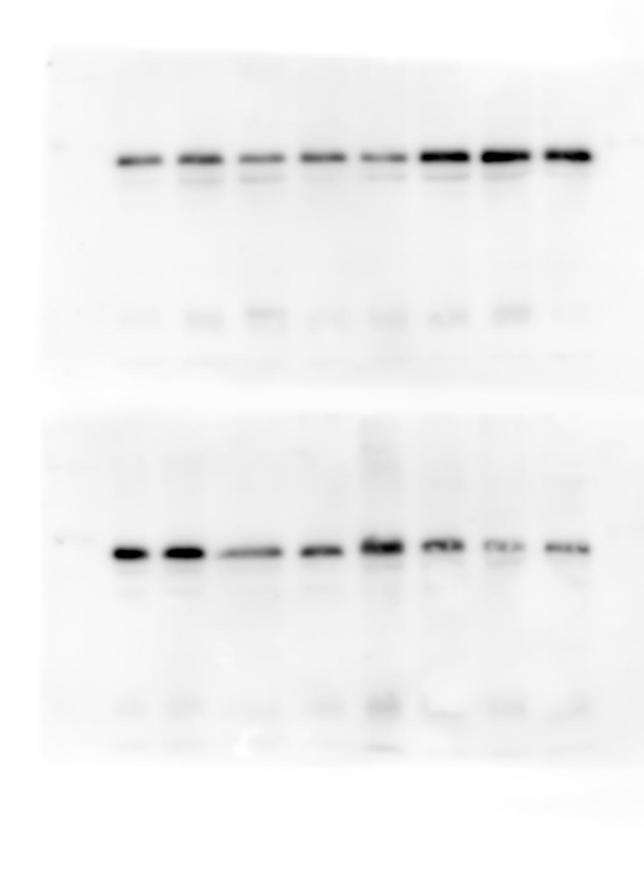

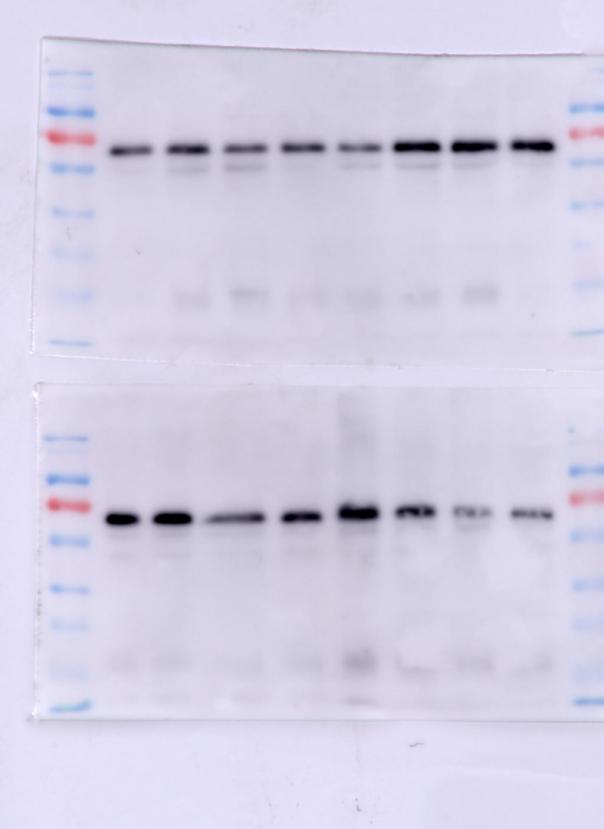


# Fig7.E-β-Tubulin（up）/GAPDH（down）


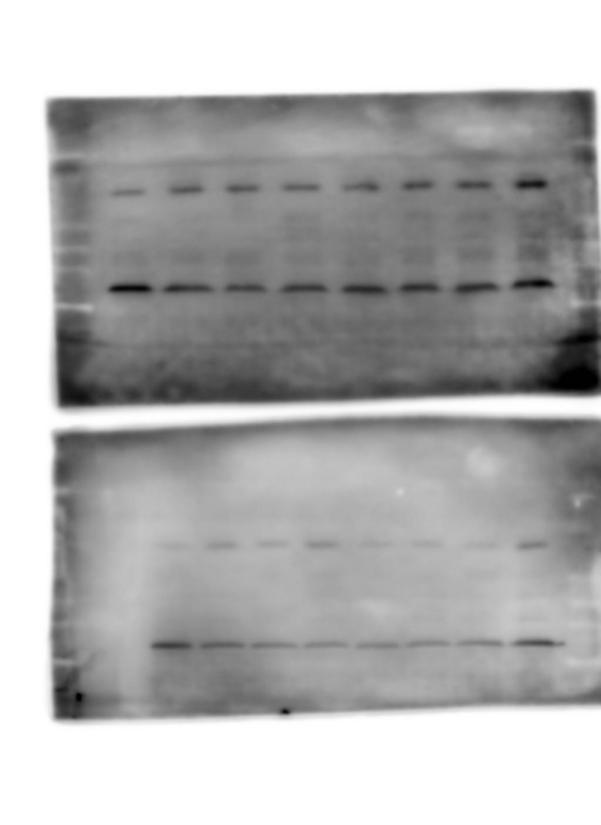

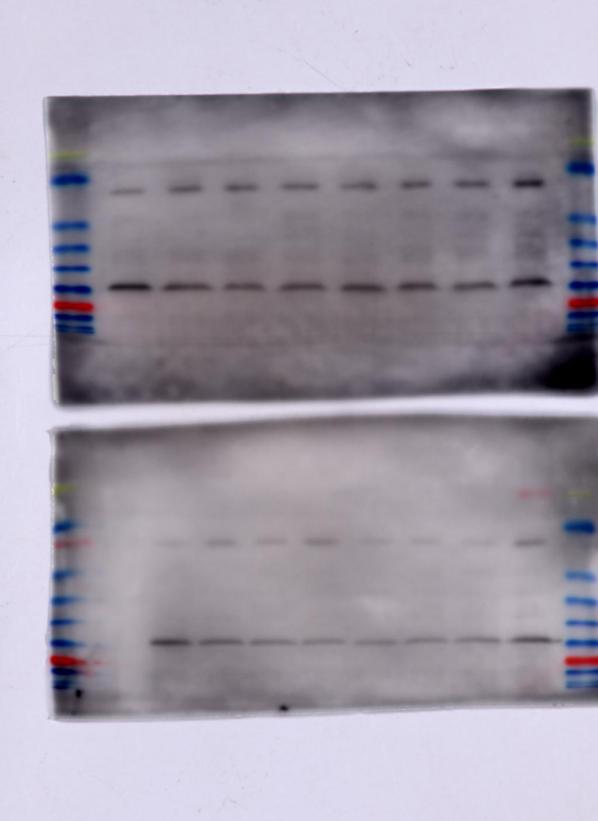


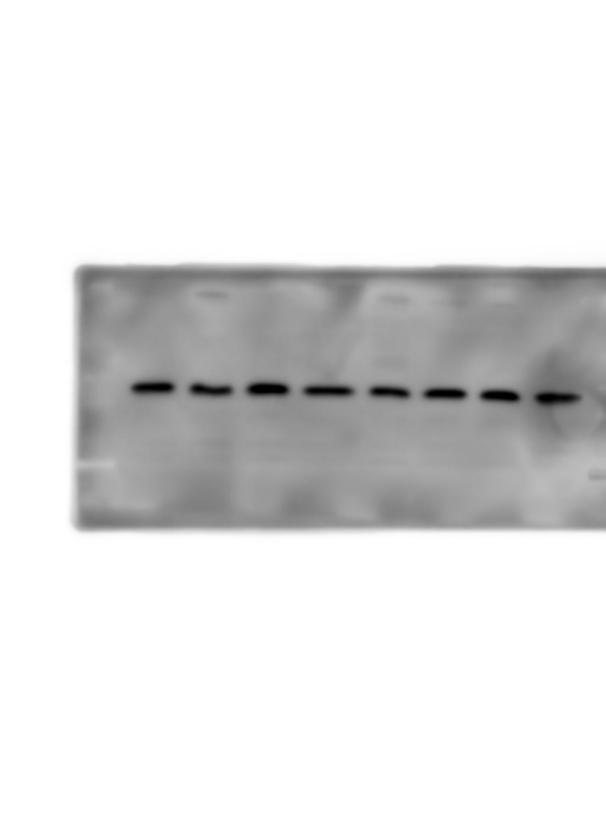

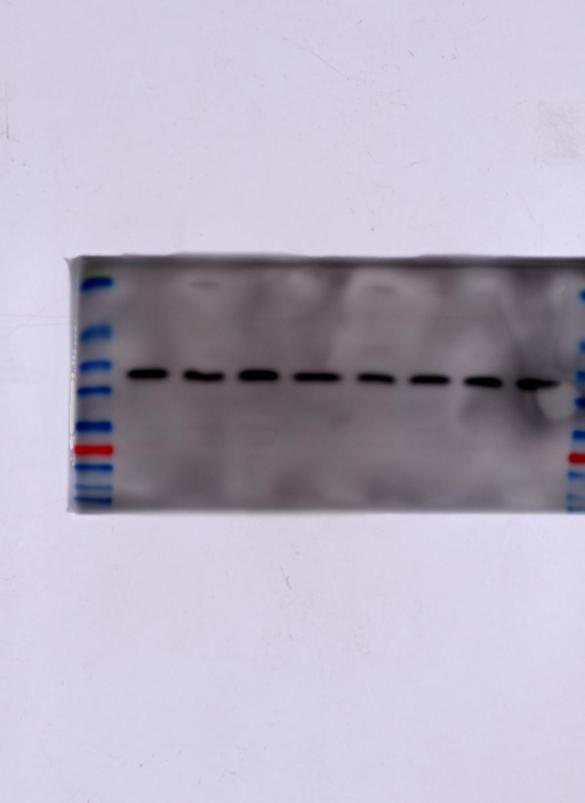


# Fig8.E-PINK1（up）/COX4（down）


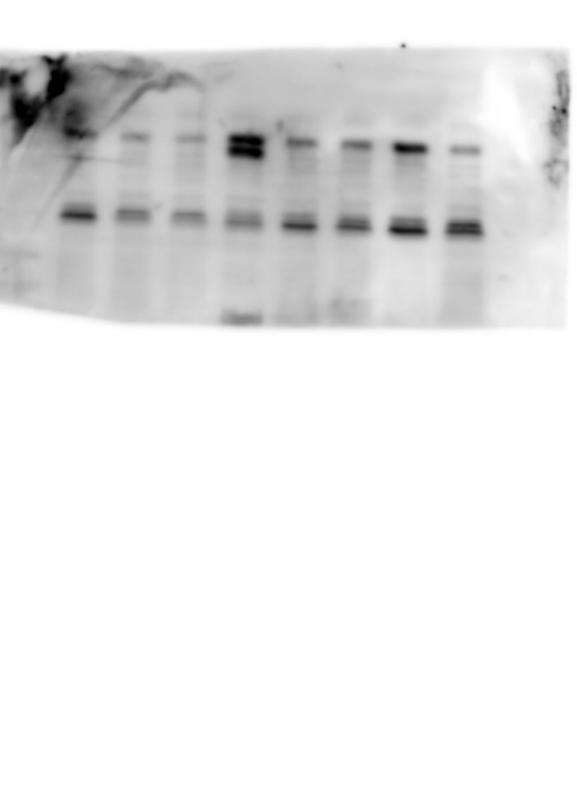

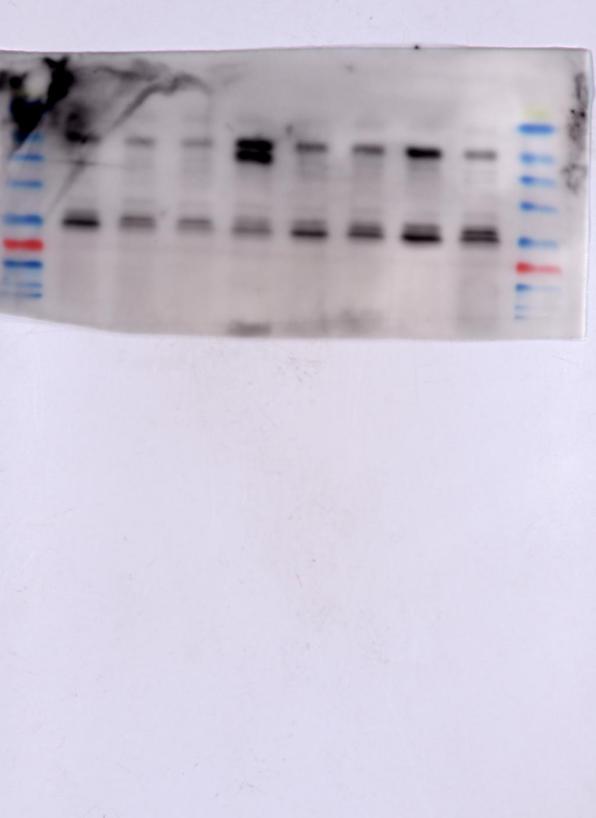


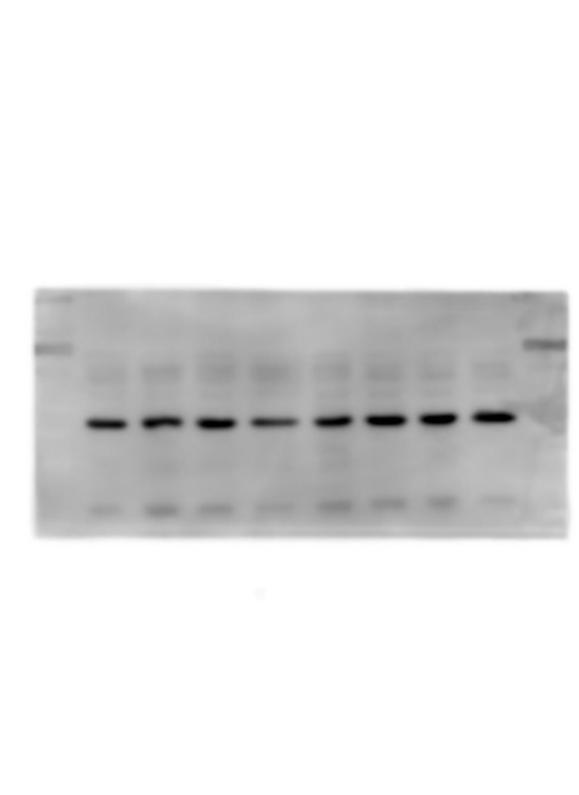

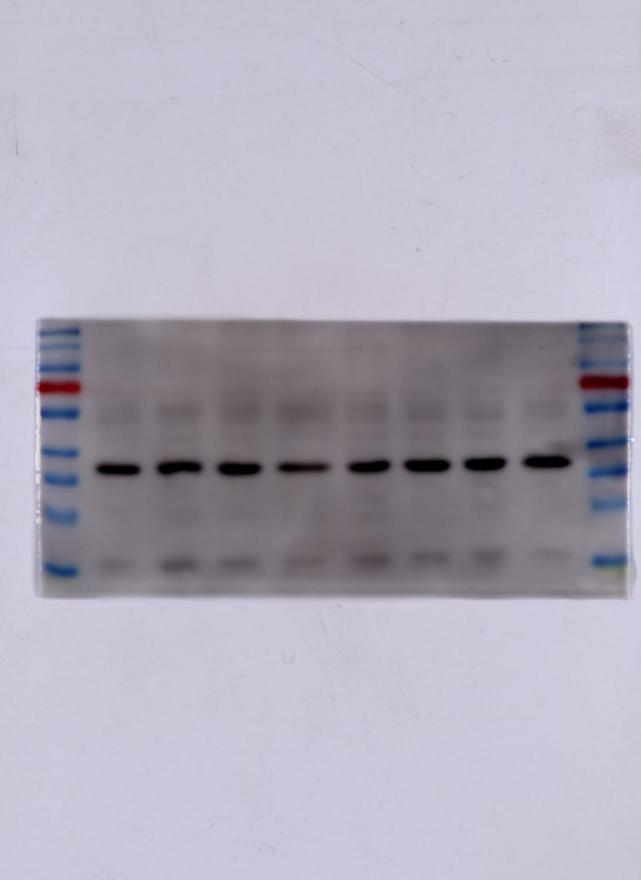


# Fig8.E-Bax（up）/Bcl2（down）


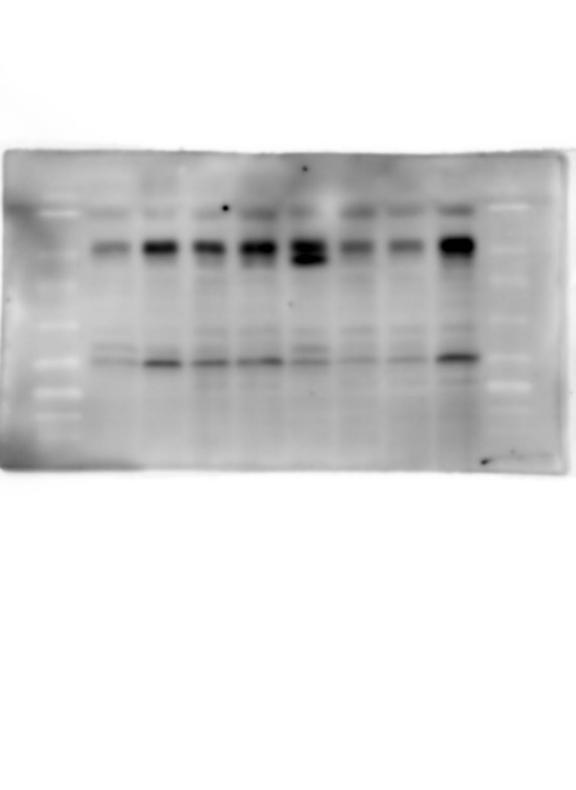

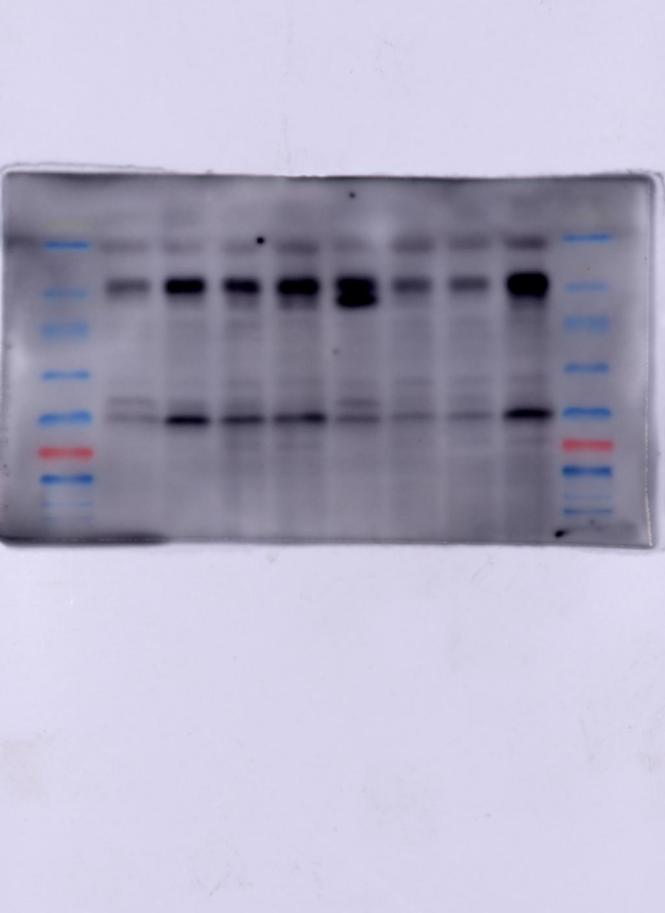


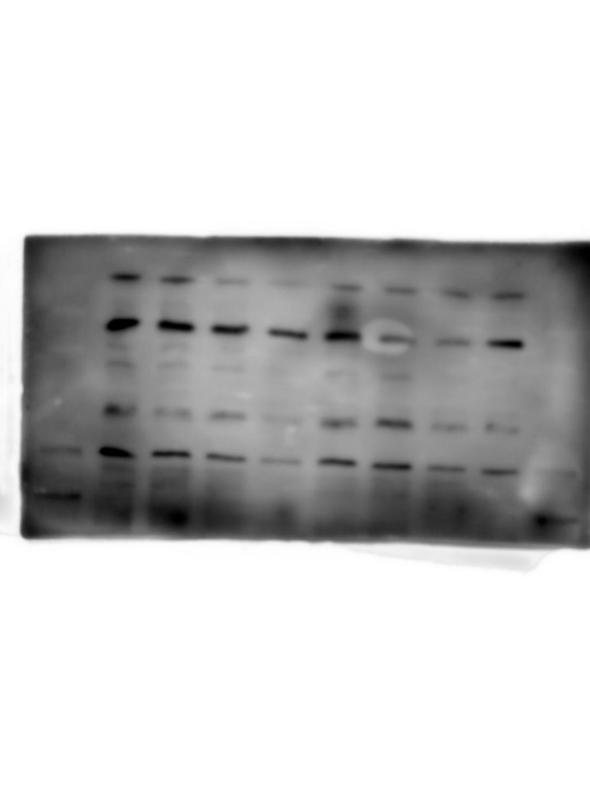

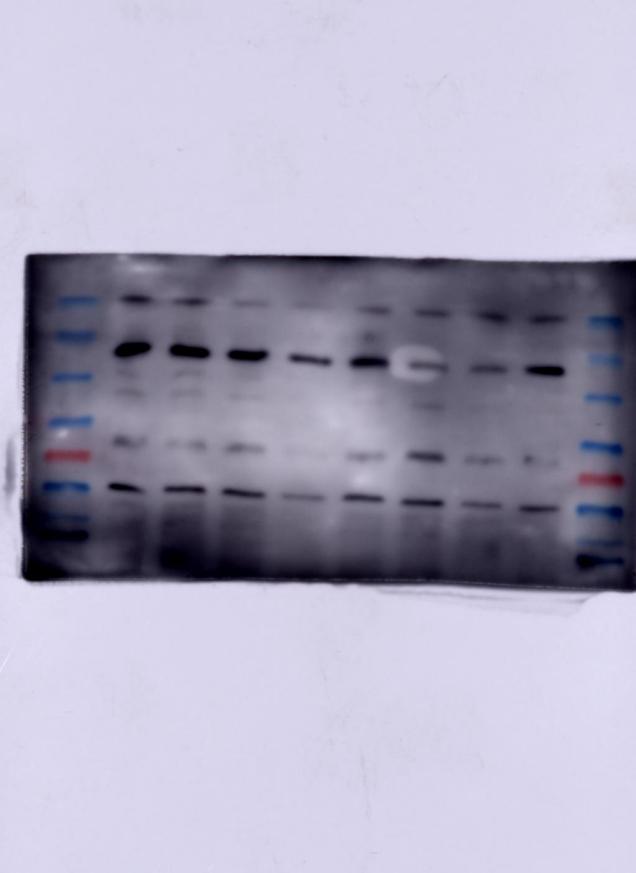


# Fig8.E-GAPDH


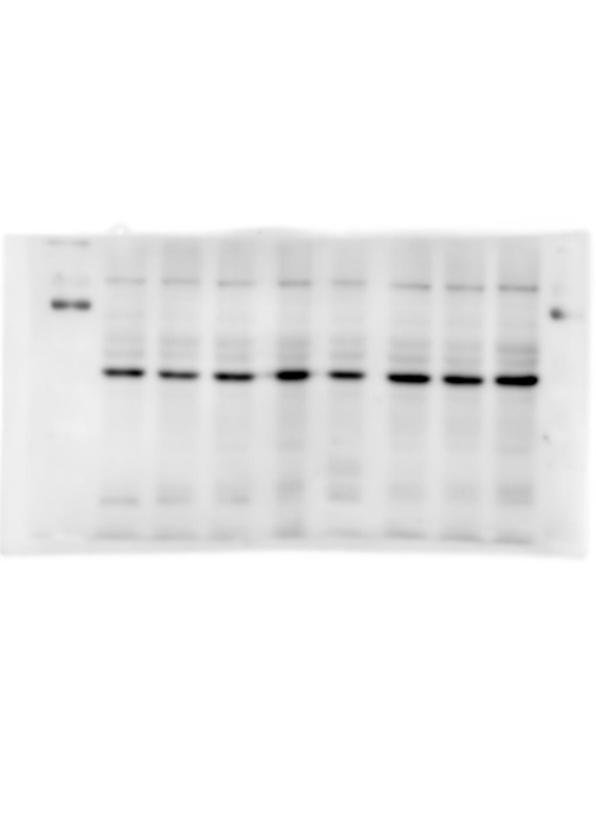

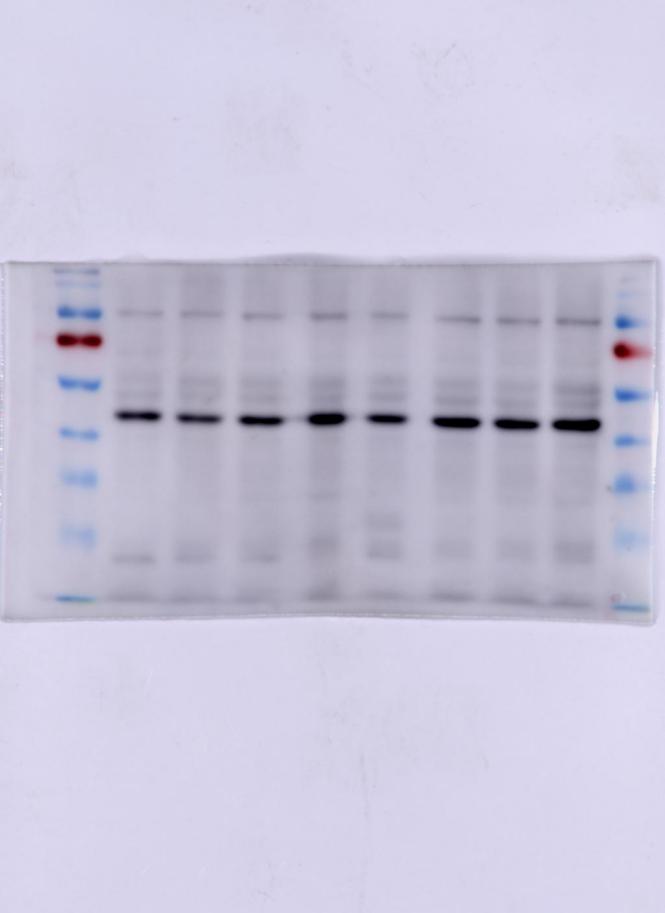


# Fig8.J-mito-Parkin（up）/TOMM20（down）


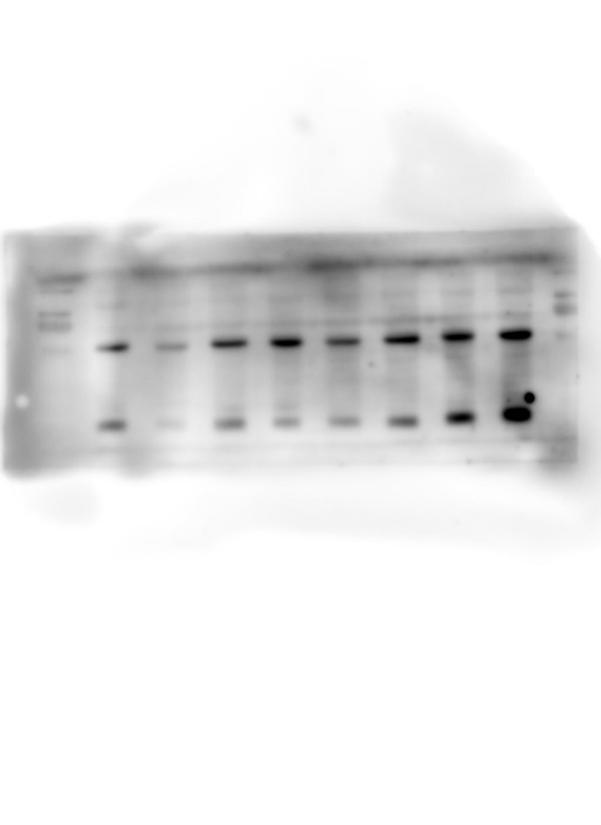

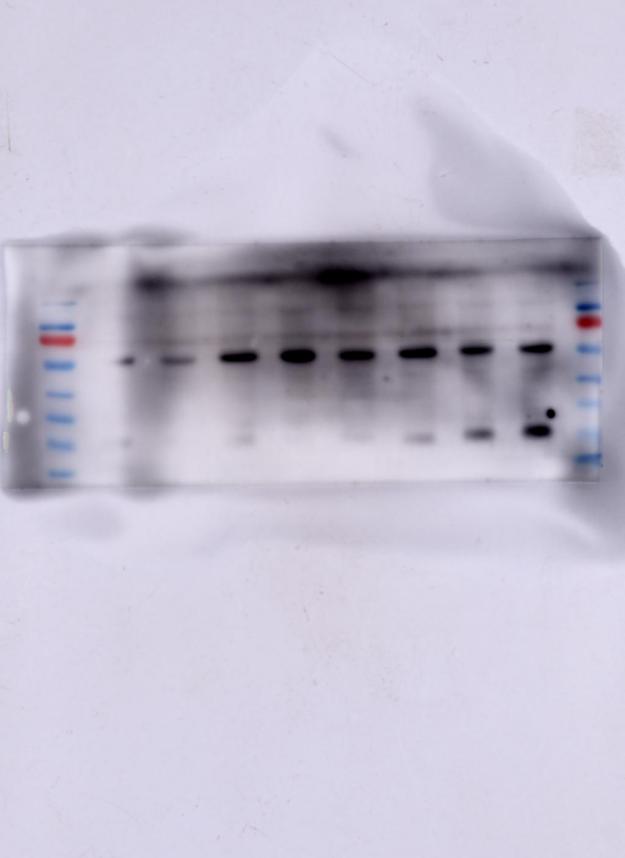


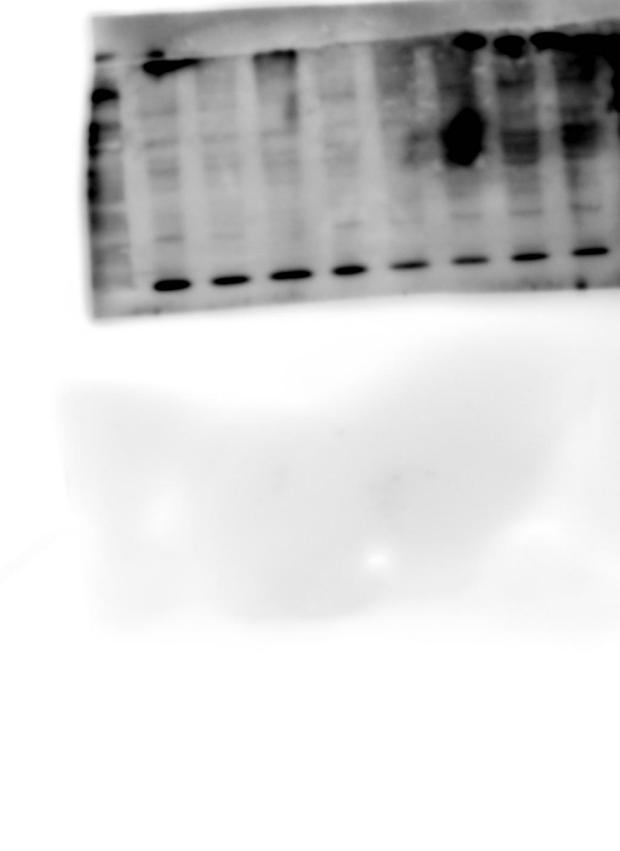

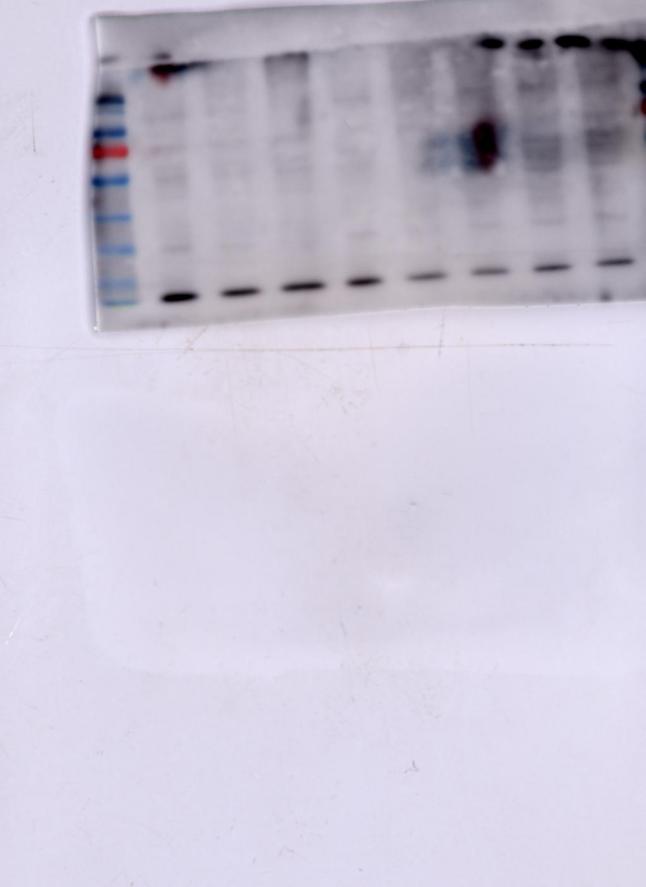


# Fig8.J-mito-COX4


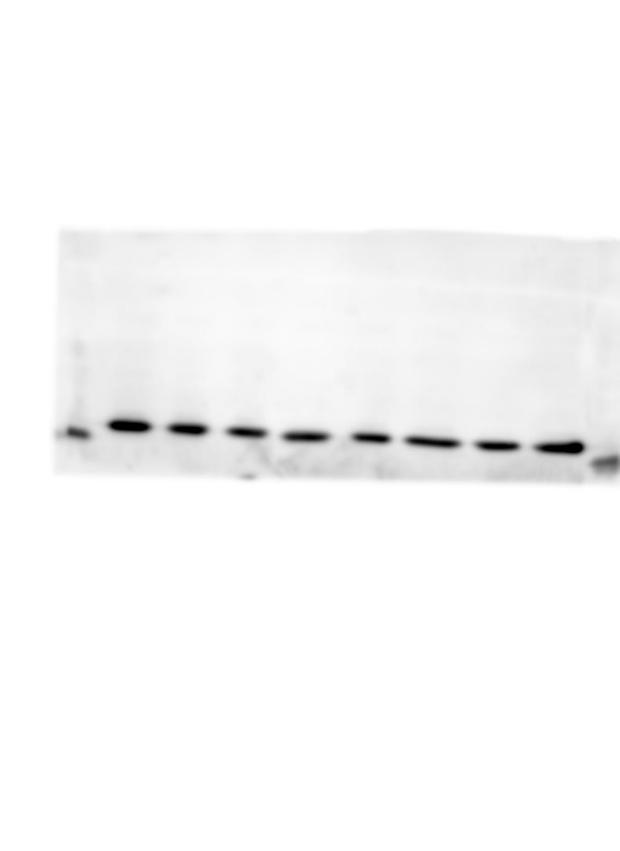

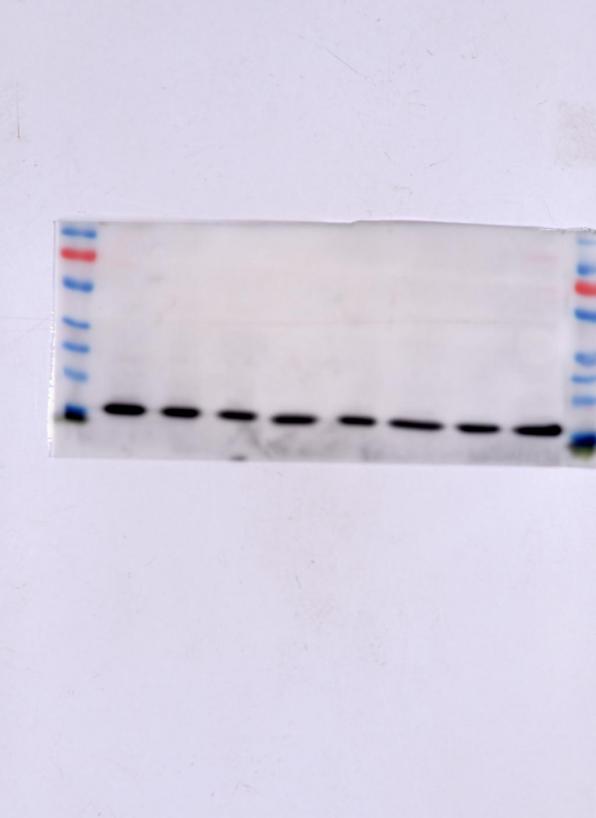


# Fig6.BE-Co-IP(FKBP-up/PPAR-γ-down)


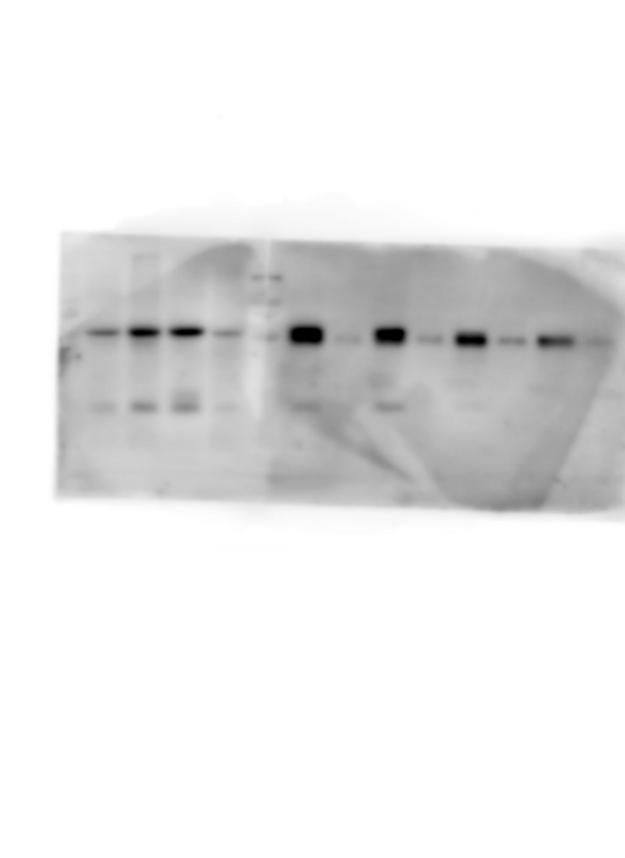

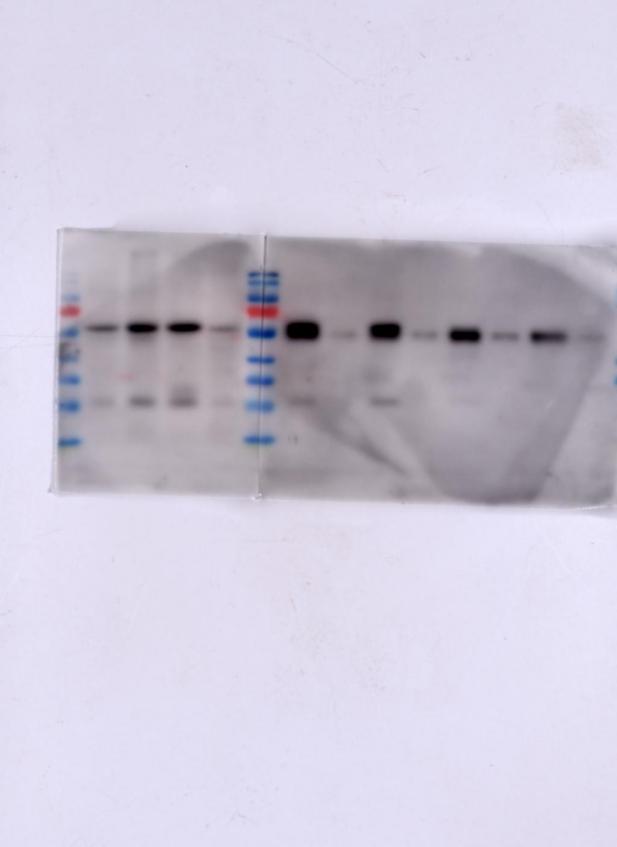


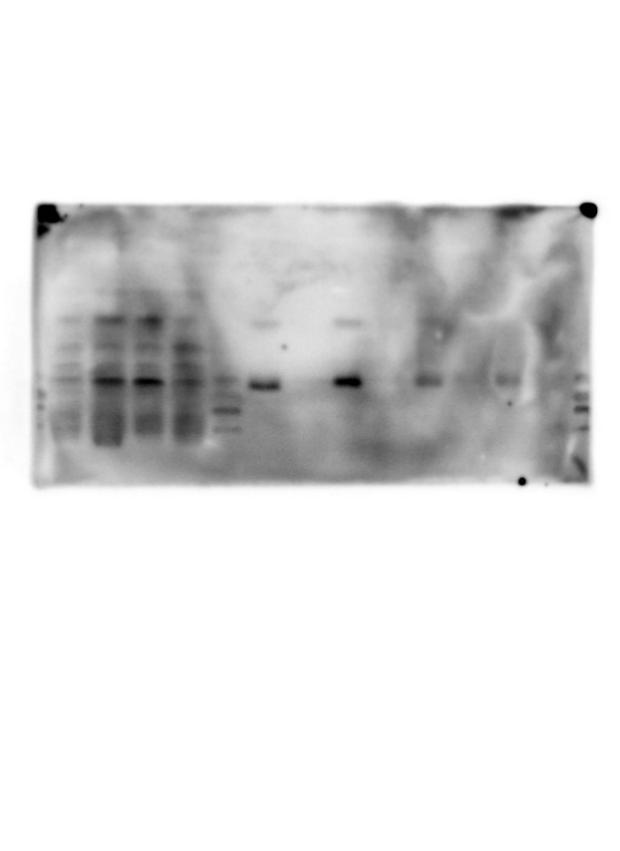

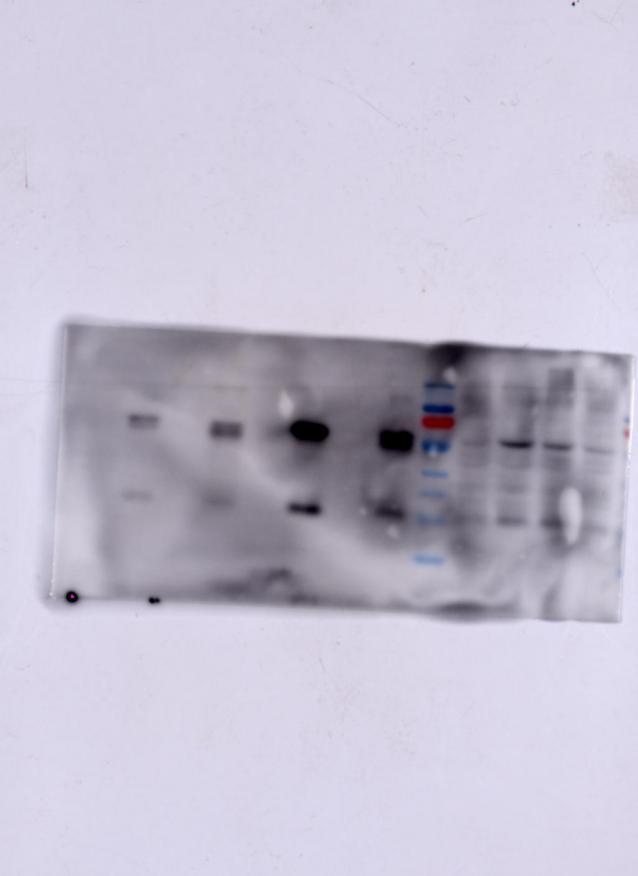


# Fig9.C-Co-IP(FKBP-up&PPAR-γ-down-PINK)


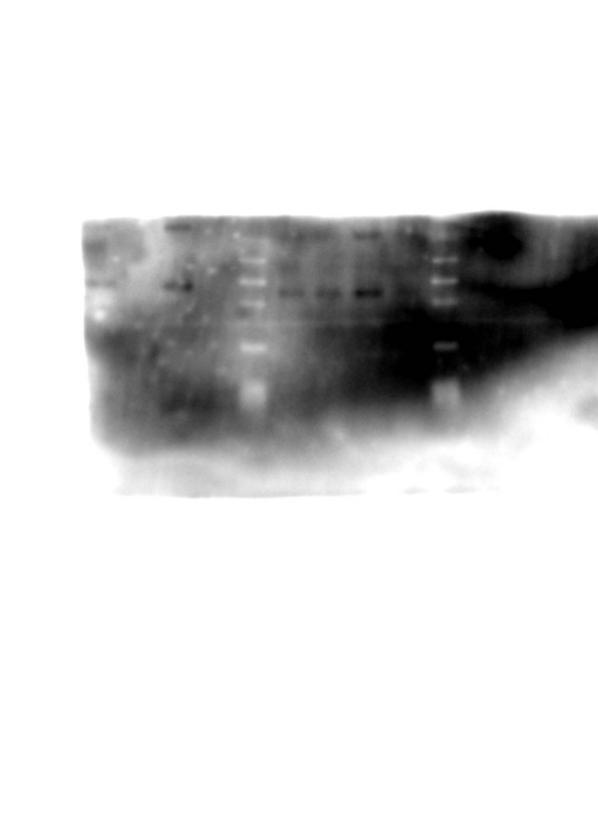

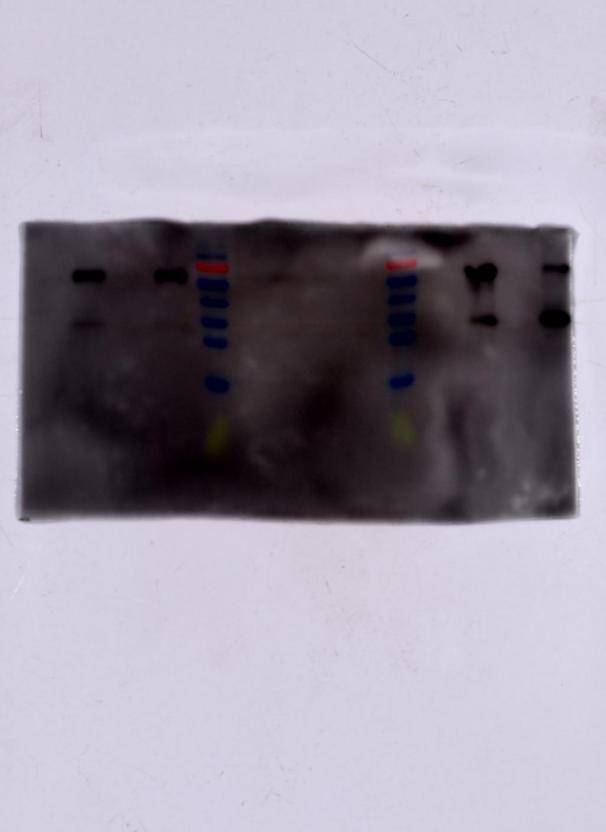


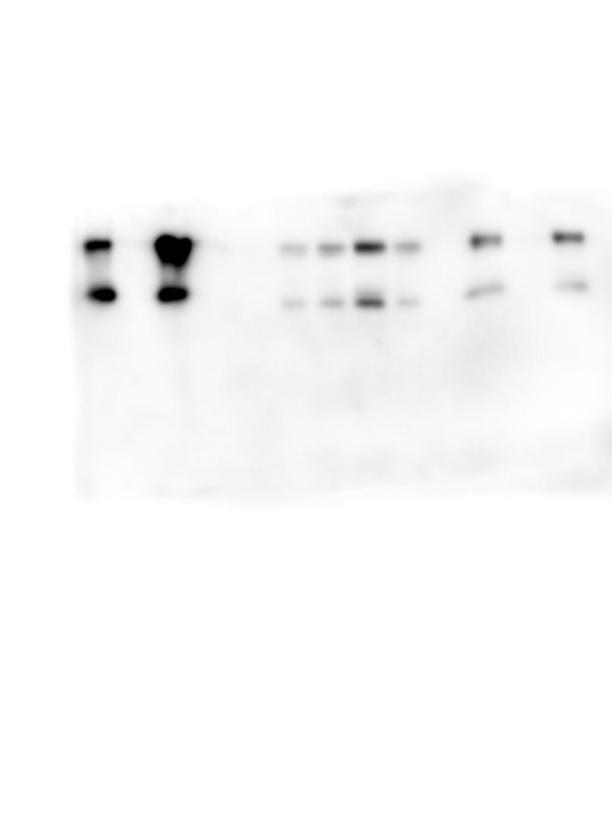

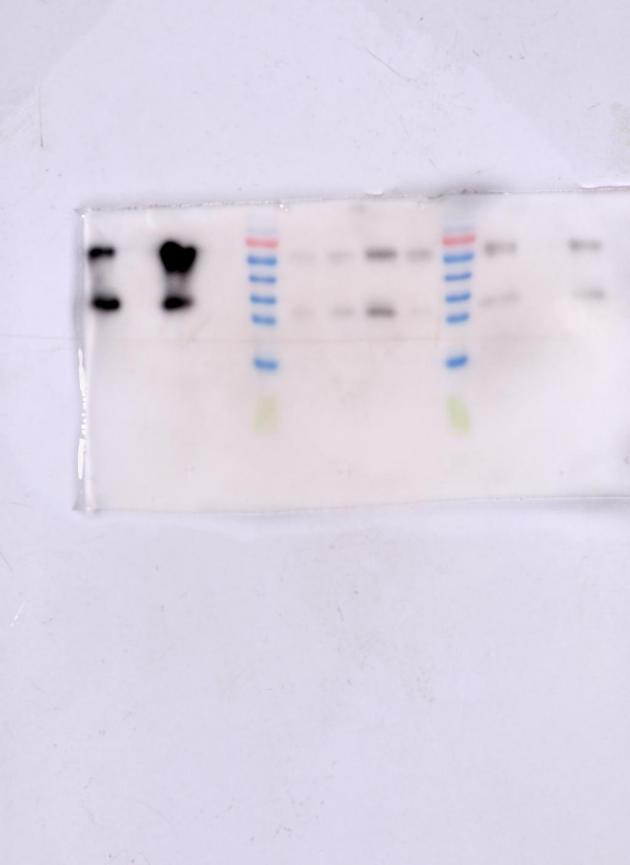


# Supplementary Figure S1A-COX-IV-β-Tubulin（From top to bottom）

#
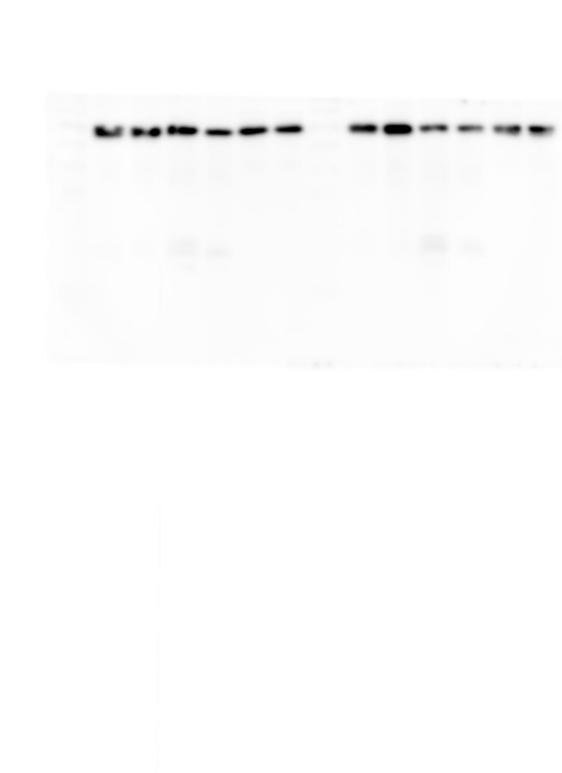

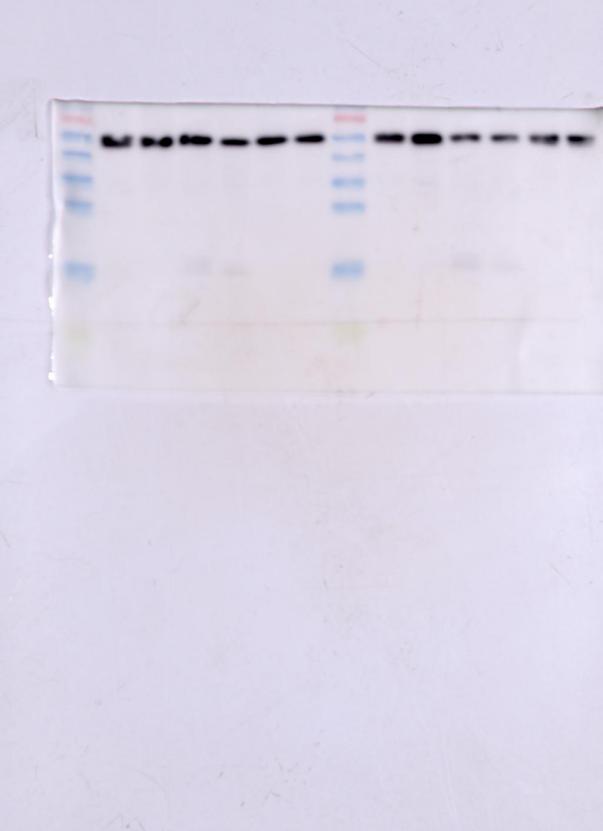


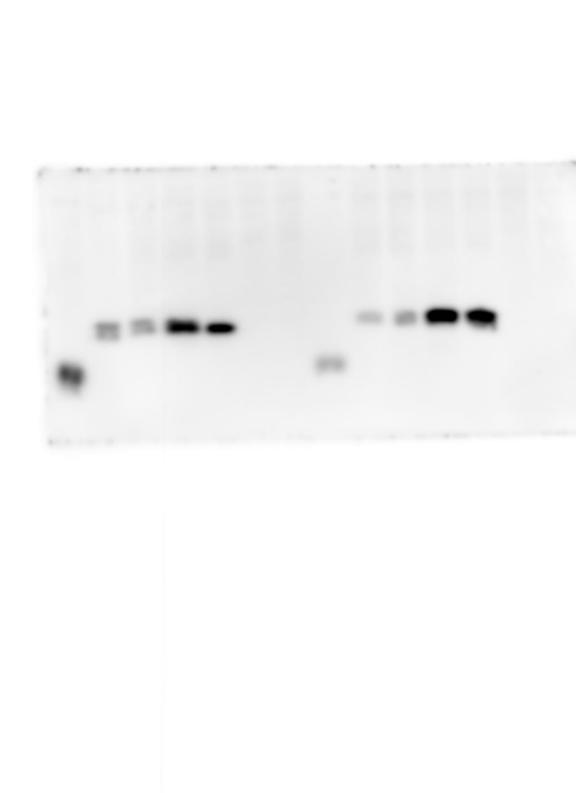

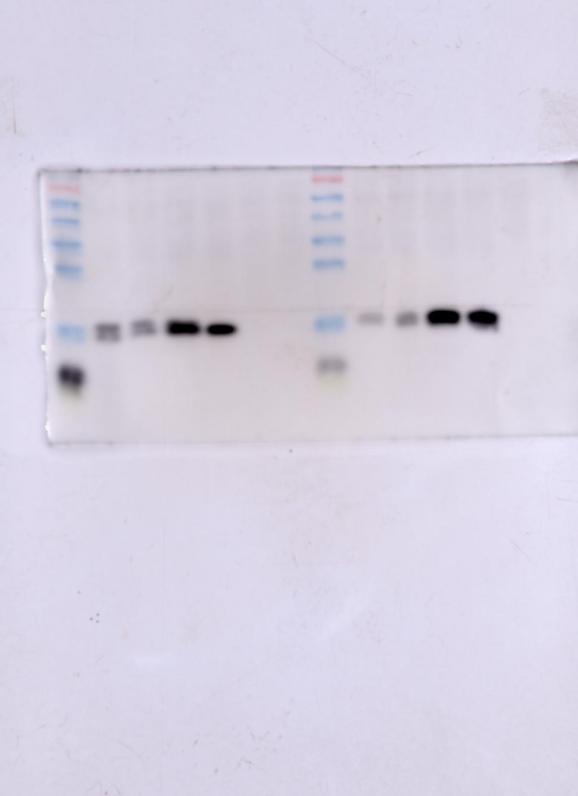


# Supplementary Figure S1B-FKBP5-GAPDH

# （From top to bottom）

#
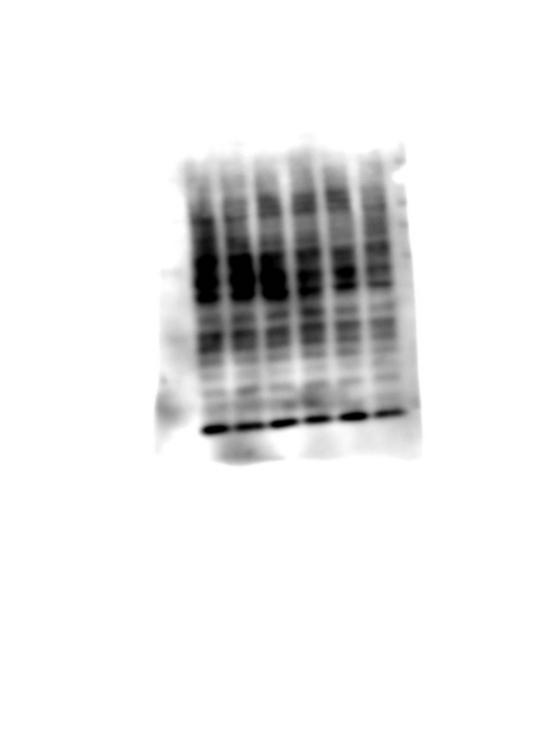

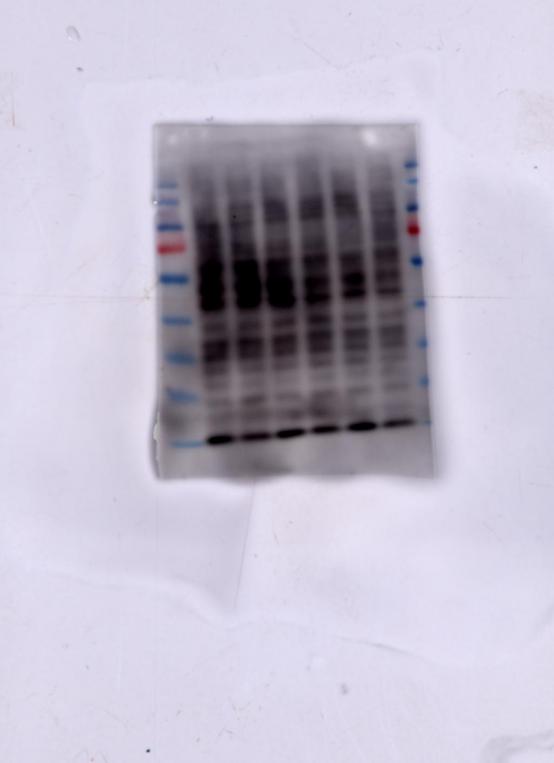


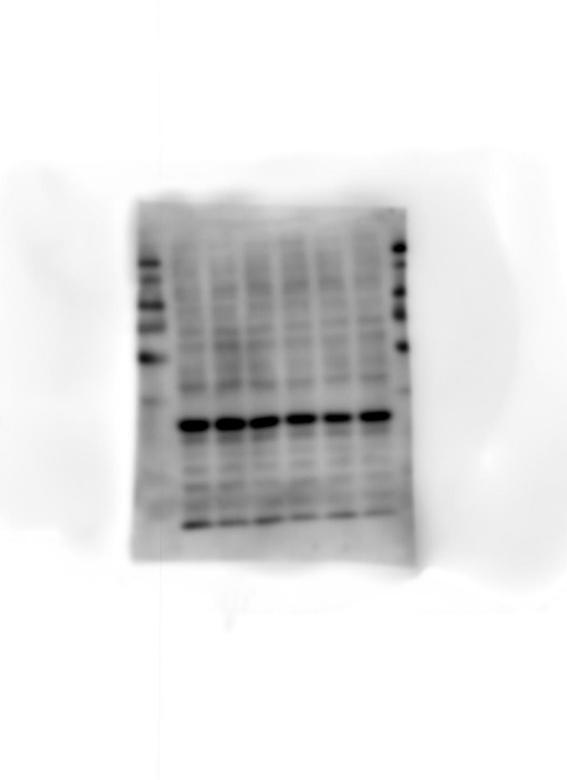

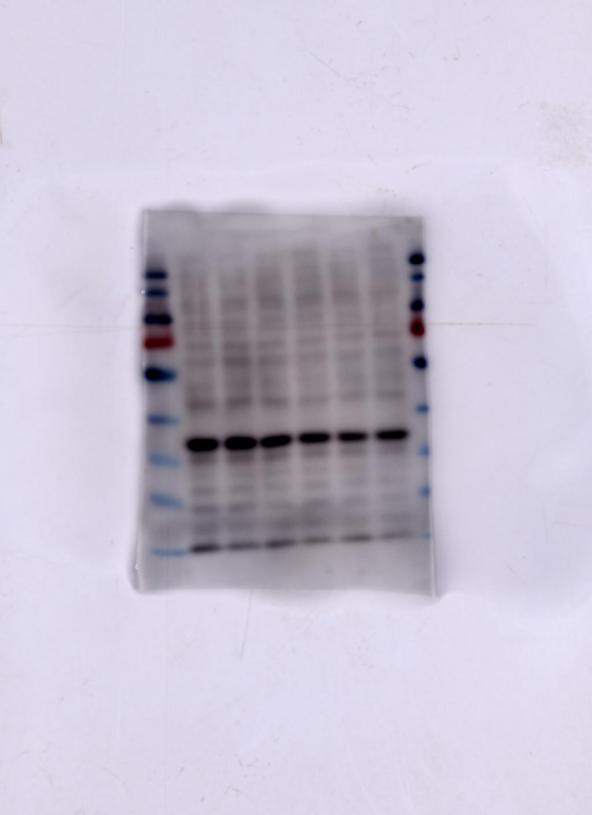


# Supplementary Figure S1C-Bcl2-GAPDH（From top to bottom）


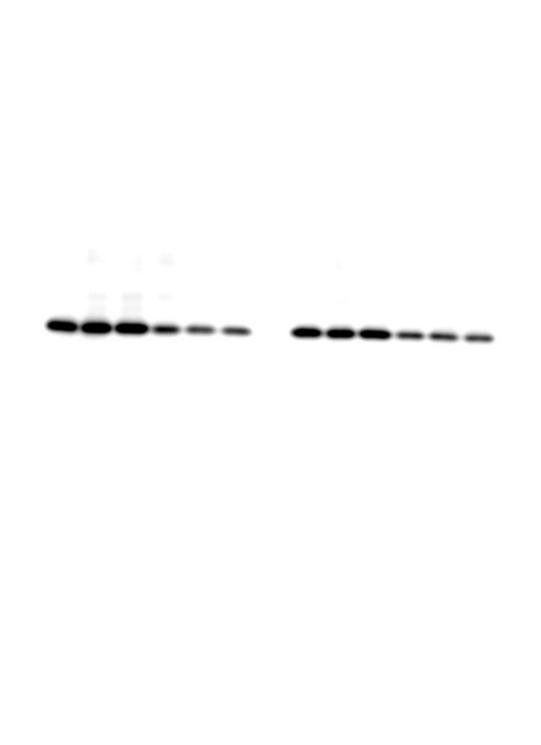

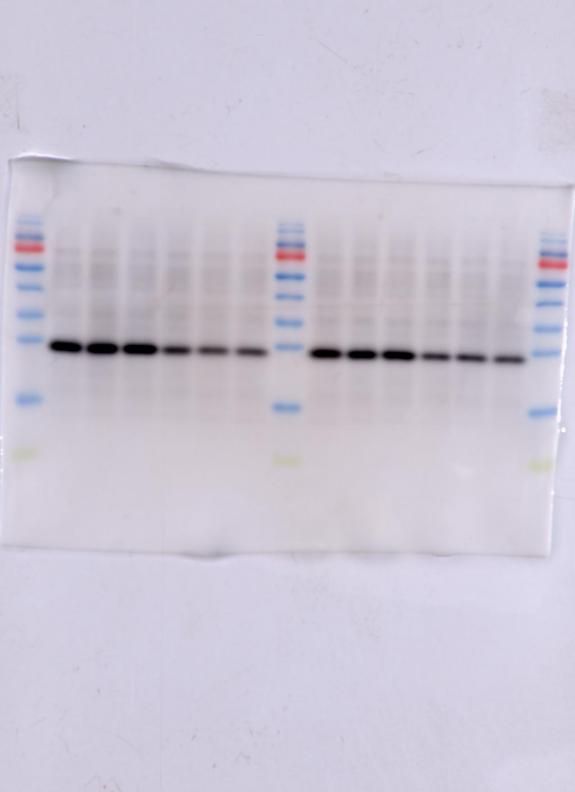


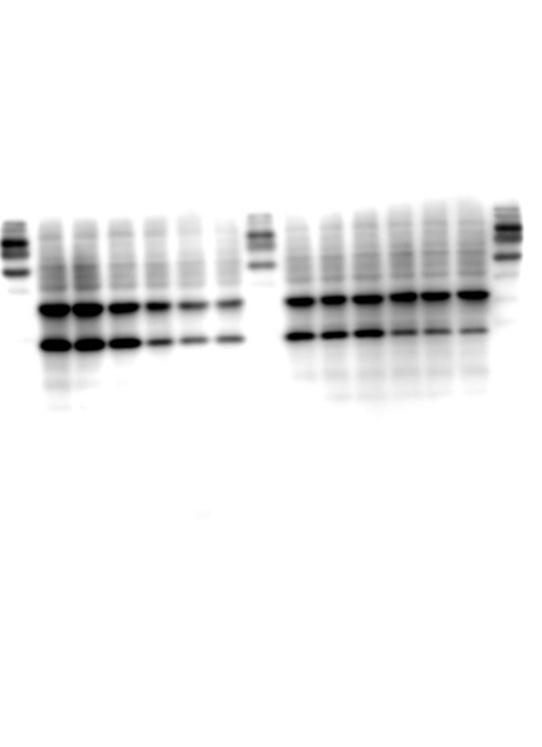

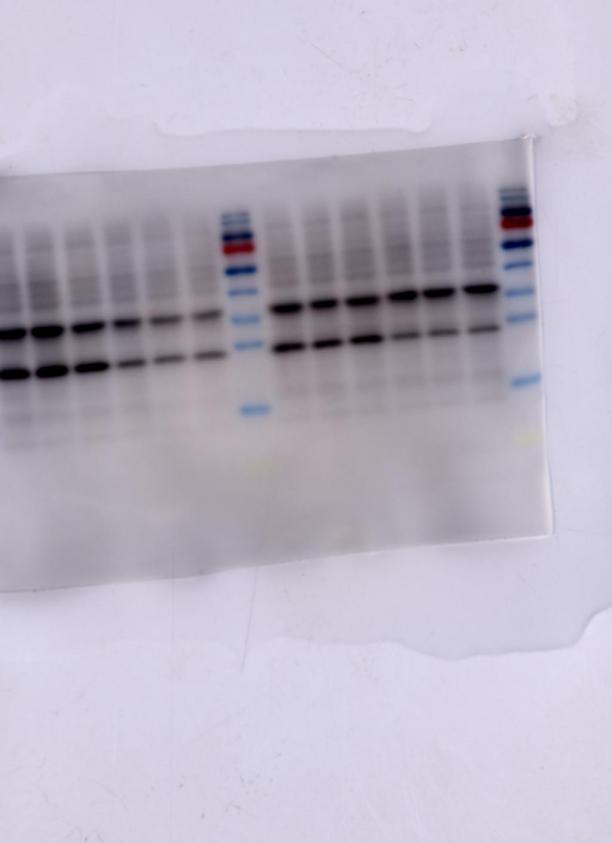


# Supplementary Figure S4C-TOMM20-Parkin-β-Tubulin（From top to bottom）


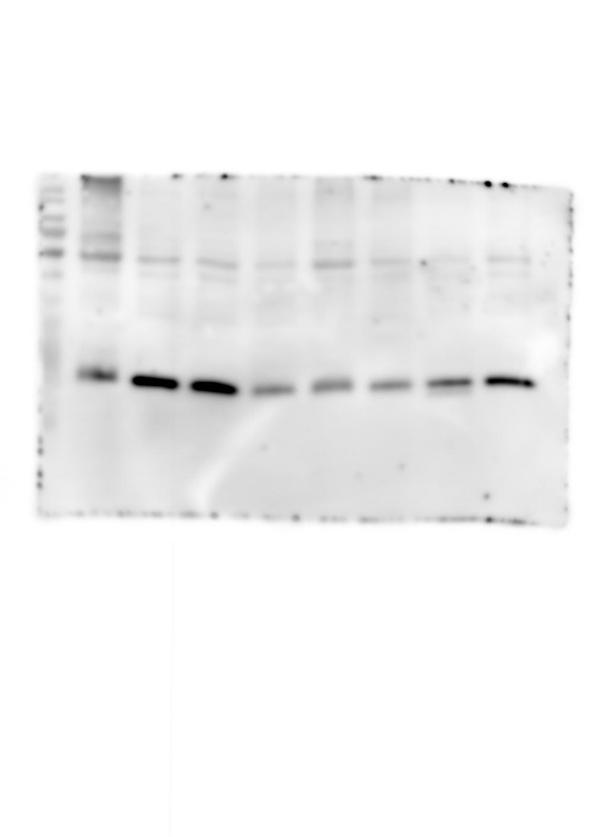

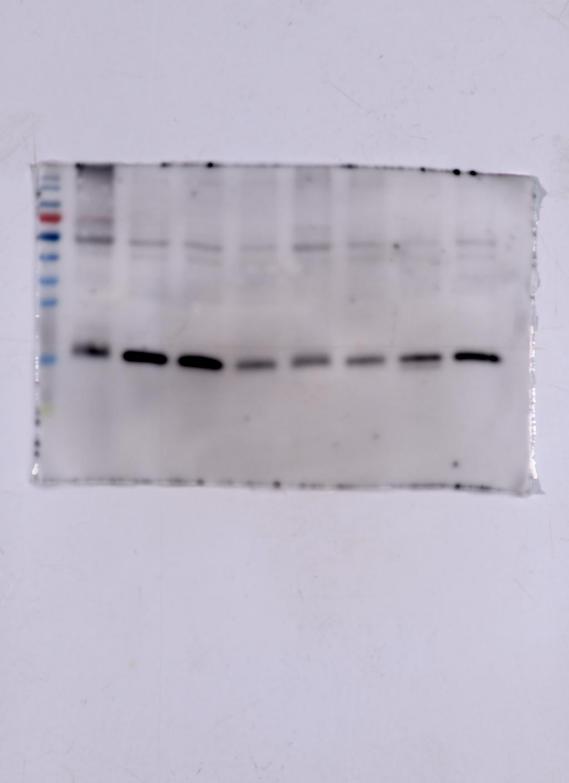


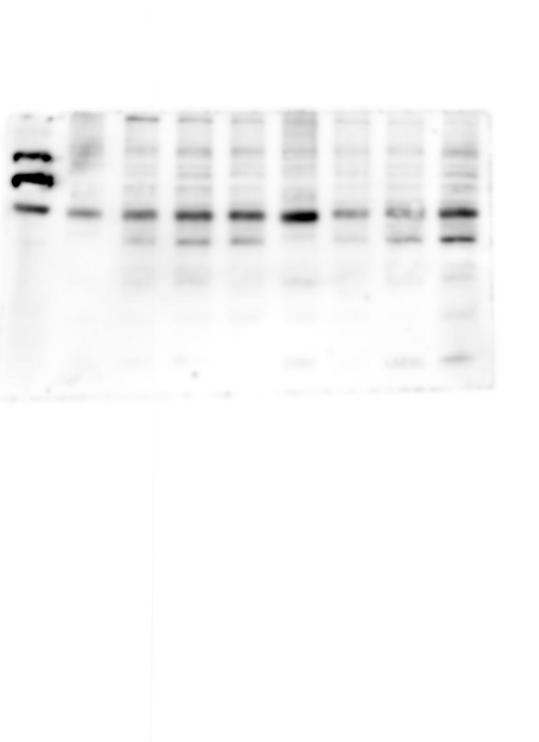

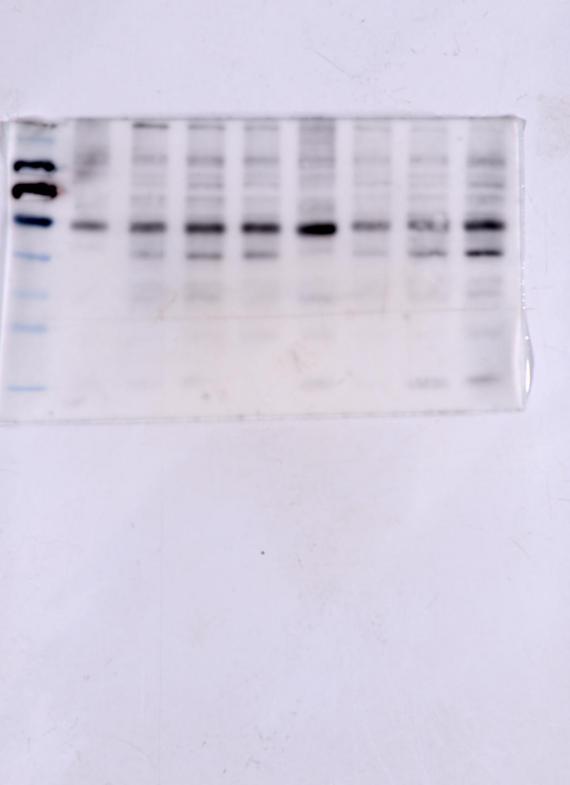

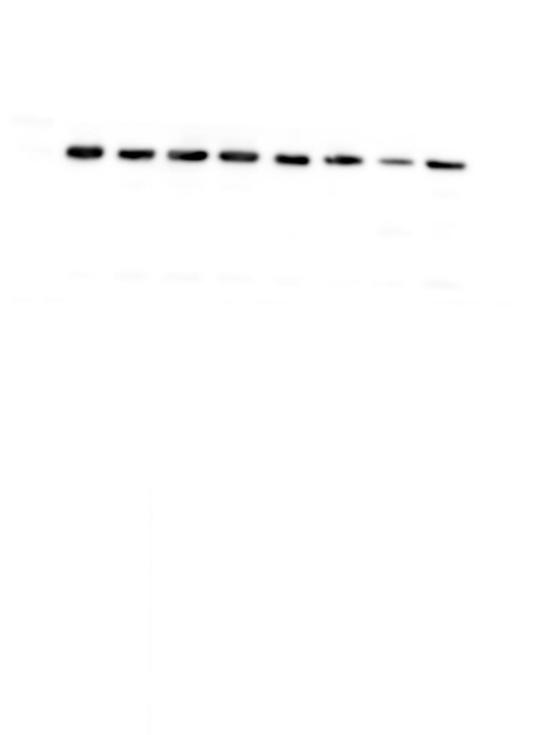

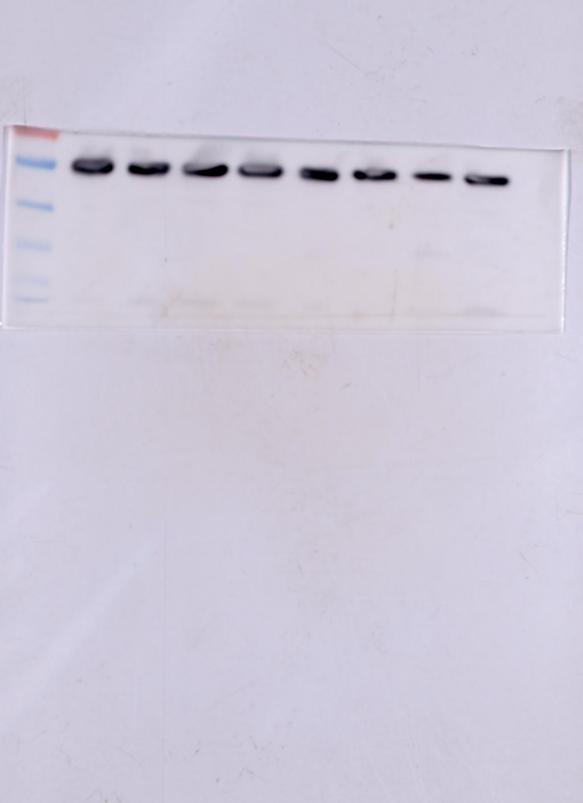

Supplement: Supplementary file 3 — Full length uncropped original western blots [file 41419_2023_6260_MOESM3_ESM.docx]
